# Investigating the effect of textural properties on CO_2_ adsorption in porous carbons via deep neural networks using various training algorithms

**DOI:** 10.1038/s41598-023-48683-4

**Published:** 2023-12-02

**Authors:** Pardis Mehrmohammadi, Ahad Ghaemi

**Affiliations:** School of Chemical, Petroleum and Gas Engineering, Iran University of Science and Technology, Tehran, 16765-193 Iran

**Keywords:** Chemical engineering, Environmental impact

## Abstract

The adsorption of carbon dioxide (CO_2_) on porous carbon materials offers a promising avenue for cost-effective CO_2_ emissions mitigation. This study investigates the impact of textural properties, particularly micropores, on CO_2_ adsorption capacity. Multilayer perceptron (MLP) neural networks were employed and trained with various algorithms to simulate CO_2_ adsorption. Study findings reveal that the Levenberg–Marquardt (LM) algorithm excels with a remarkable mean squared error (MSE) of 2.6293E−5, indicating its superior accuracy. Efficiency analysis demonstrates that the scaled conjugate gradient (SCG) algorithm boasts the shortest runtime, while the Broyden–Fletcher–Goldfarb–Shanno (BFGS) algorithm requires the longest. The LM algorithm also converges with the fewest epochs, highlighting its efficiency. Furthermore, optimization identifies an optimal radial basis function (RBF) network configuration with nine neurons in the hidden layer and an MSE of 9.840E−5. Evaluation with new data points shows that the MLP network using the LM and bayesian regularization (BR) algorithms achieves the highest accuracy. This research underscores the potential of MLP deep neural networks with the LM and BR training algorithms for process simulation and provides insights into the pressure-dependent behavior of CO_2_ adsorption. These findings contribute to our understanding of CO_2_ adsorption processes and offer valuable insights for predicting gas adsorption behavior, especially in scenarios where micropores dominate at lower pressures and mesopores at higher pressures.

## Introduction

Recent years have seen a sharp rise in the total amount of carbon dioxide in the atmosphere, causing numerous environmental concerns^1^. CO_2_ has been identified as the predominant greenhouse gas in the atmosphere and as the primary cause of climate change^2–4^. Human activities, including the combustion of fossil fuels, agricultural practices, and industrial processes, are the primary sources of CO_2_ emissions into the atmosphere^5^. Therefore, efficient carbon capture and storage techniques are crucial for environmental protection^6^.

Carbon dioxide capture technology can be categorized into five primary classes, which are cryogenic distillation^7–10^, membrane^11–14^, absorption^15–18^, and adsorption^19–22^. The implementation of membrane, cryogenic, and absorption technologies has encountered significant obstacles due to their high cost^23^, environmental concerns^24^, and substantial energy consumption^25^. In contrast, adsorption technology is not only recognized as an environmentally sustainable method, but it also has a notable characteristic of consuming less energy during the separation of carbon dioxide, hence demonstrating high selectivity and efficiency^26^. Recently, carbon capture and storage (CCS) has garnered considerable attention due to its applications^27^.

Commercial adsorbents used in CO_2_ adsorption methods include porous carbon^28–30^, zeolites^31–33^, and metal–organic frameworks (MOFs)^34–36^. Despite the high CO_2_ adsorption capacities of zeolites and MOFs, these extremely hydrophilic materials' adsorption capacities might drastically decrease in the presence of water^37,38^. However, new MOFs have recently been synthesized that are stable against water^39^. In contrast, porous carbon is hydrophobic, has a large surface area, and demonstrates exceptional thermal and chemical stability. In addition, it is simple to prepare, has a pore size that can be controlled, and is inexpensive. The structure and porosity of the resulting porous carbon are determined by the kind of carbon precursor and preparation technique used^40^.

Numerous investigations have been allocated to the development of porous carbons with a high pore volume and specific surface aim to enhance CO_2_ adsorption^29,41^. The main contributor to CO_2_ adsorption is now generally acknowledged to be narrow micropores; nevertheless, the precise link between pore structure and CO_2_ adsorption is still unknown^42–44^. There have previously been phenomenological efforts to investigate the effect of textural property on CO_2_ adsorption capacity. Durá et al., for example, suggested a regression equation to correlate CO_2_ adsorption with the volume of micropores mesopores^45^. As shown in Eq. (1), CO_2_ adsorption was predicted using a partial least square regression approach. Although this regression equation has the ability to produce a reasonable estimate, the regression polynomial method makes it challenging to obtain an ideal empirical formula. In addition, this regression equation cannot be learned and developed as the database grows^46^. Importantly, according to Eq. (1), ordered mesoporous carbon without micropores ought to have almost no capacity to adsorb CO_2_, which is preposterous in some instances. In addition, the equation is derived from a small sample size, with only 12 varieties of porous carbon examined in their research. All of these samples were at a pressure of 5 bar and a temperature of 25 °C, so the operating conditions of the adsorbers that use this relationship are restricted to these conditions.

1 $$ M_{ads}^{{CO_{2} }} = 0.095 + 2.10 \times V_{micro} + 3.51 \times V_{micro} \times V_{meso} $$

So far, quantitative investigations utilizing the known textural parameters of porous carbons have successfully enabled precise forecasts of CO_2_ adsorption. Shen et al.^47^ developed hierarchical porous activated carbon fibers, which exhibit faster adsorption rates and higher capacity when compared to pure carbon materials. Xia et al.'s^48^ study shows high surface area, microporous, nitrogenous properties, and zeolite pattern carbons have the highest CO_2_ adsorption capacity. Casco et al.^49^ observed that activated carbons derived from crude oil and potassium hydroxide display exceptional CO_2_ adsorption performance in atmospheric and high-pressure conditions. The optimal carbon structure is contingent upon the specific application, with narrow micropores exerting control and ensuring high delivery capacity. Based on the review of existing literature, it is notable that there is a lack of a comprehensive and highly accurate model that can effectively predict the amount of carbon dioxide adsorbed by the textural properties of adsorbents and the operating conditions. Deep learning (DL) models are frequently regarded as a robust approach for modeling purposes, owing to their capacity to yield highly accurate predictions. However, it is notable that these models cannot offer a quantitative analysis of each input, as they operate based on the concept of black boxes.

In recent years, deep learning has exhibited significant potential in addressing a multitude of material research-related challenges^50–53^. Specifically, research endeavors have been undertaken within the domain of neural network modeling and simulation to study carbon dioxide adsorption processes. Dashti et al.^54^ created an MLP network to assess the adsorption of pure gases on activated carbon and zeolite-5A. They developed accurate models using input parameters such as temperature, pressure, pore size, and kinetic diameter. To optimize the model, various hidden layers were constructed, and the dataset's AARD% value was used to evaluate performance. Fotoohi et al.^55^ used four two-dimensional equations of state to assess the adsorption of pure and binary gases onto activated carbons. They applied the LM algorithm for model learning and the ANN method for prediction. Compared to two-dimensional state equations, the optimal architecture was 1-6-7 for pure gas adsorption and 1-7-9 for binary gas adsorption, with a higher precision. Iraji et al.^56^ surveyed the adsorption of CO_2_ and SO_2_ on modified carbon nanotubes. They proposed an MLP neural network model with three hidden layers and 10 neurons, which was trained with the LM training algorithm. Lepri et al.^57^ developed reduced-order PSA models using artificial neural networks that demonstrated excellent agreement between ANN and simulation results, allowing for their implementation in optimization environments for PSA cycle synthesis. Meng et al.^58^ explored the adsorption of supercritical CO_2_ and CH_4_ on coal using conventional isotherm models. In addition, they proposed a novel machine learning model based on a 15-neuron neural network. Zhang et al.^44^ predicted the CO_2_ adsorption capacity of porous carbons using the DL algorithm with five input parameters: specific surface area, micropore volume, mesopore volume, temperature, and pressure. Also, the highest prediction accuracy was achieved when all three textural properties were used. At low pressure, microporous porous carbon adsorbs more CO_2_, whereas hierarchical porous carbon adsorbs more at high pressure. However, the DNN model presented in this research was prepared by MATLAB toolbox, which due to its limitations, such as creating a model with only two hidden layers with an equal number of neurons, the impossibility of changing the default activation functions for the layers cannot be an optimal model.

Table 1 presents a summary of further research on the ANN models for simulating CO_2_ adsorption processes. It can be seen that the diversity of training algorithms applied to train MLP neural networks in CO_2_ adsorption processes is comparatively limited when compared to other processes, with Levenberg–Marquardt (LM) and Bayesian regularization (BR) algorithms being the primary choices.

**Table 1:** Some studies carried out in the application of neural networks on CO_2_ adsorption.

Author	Network inputs	Structure	Activation function	Training algorithm	R or R^2^	References
Rostami et al.	CO_2_ partial pressure and temperature	[9 4 1]	Tansig/purelin	LM	0.9999	^59^
Kareem et al.	Pressure and temperature (Zeolite X13 at 50 C)	[2 7 1]	Not mentioned	LM	0.9970	^60^
	Pressure and temperature (Zeolite X13 at 70 C)				0.9928	
	Pressure and temperature (Zeolite A5 at 50 C)	[2 11 1]			0.9960	
	Pressure and temperature (Zeolite A5 at 70 C)				0.9909	
Mashhadimoslem et al.	Precursors, activators, pyrolysis temperature, drop volume, adsorption pressure and adsorption temperature	[6 45 1]	Logsig/purelin	BR	0.9716	^61^
Khoshraftar and Ghaemi	Pressure and temperature	[2 10 1]	Tansig/purelin	LM	0.9998	^62^
Barki et el.	Specific surface area, micropore volume, temperature and pressure	[4 16 1]	Tansig/Tansig/purelin	BFGS	0.9965	^63^
Torkashvand et al.	Adsorption time, crosslinker ratio and adsorbent synthesis time	[3 15 10 1]	Tansig/purelin	BR	0.9815	^64^
Moradi et al.	Adsorption time, synthesis time, crosslinker ratio, temperature and pressure	[6 10 8 7 1]	Tansig/purelin	BR	0.999	^65^

To fill the aforementioned gaps, MLP neural network and, for the first time, RBF were used to model and simulate 1345 collected data representing more than 200 distinct porous carbon adsorbents to predict the amount of CO_2_ adsorbed. Inputs for these models included adsorbent textural properties such as BET surface area, mesopore volume, micropore volume, and temperature and pressure as operating conditions. According to the literature, the impact of textural properties and adsorption conditions on CO_2_ adsorption are not independent; therefore, the Pearson correlation coefficient analysis was used to investigate the primary linear relationships between any two variables as a preliminary step. In the MLP neural network, 13 distinct training algorithms and four activation function combinations of hidden layers were applied to each algorithm to optimize the network. The accuracy, run duration, and number of epochs are then used as criteria for comparing the models in order to choose the most optimal MLP model. The present study conducted a thorough evaluation of the factors that influence CO_2_ adsorption, and it also highlighted the gap in prior research by addressing the lack of relevant analysis in previous studies. This evaluation was based on the results obtained from a simulation, highlighting the significance of understanding the impact of numerous factors on the adsorption process.

## Data gathering and preparation

More than 150 papers were screened to compile data for this study. The data used for modeling and simulation was selected from literature containing over 200 adsorbents operating at various temperatures and pressures. Some of these data were collected by Zhang et al.^44^ but this study added 325 new data to their collection. The BET surface, mesopore volume, micropore volume, temperature, pressure, and the amount of carbon dioxide adsorbed are presented in Table 2 acquiring a precise neural network model requires a large quantity of high-quality data. A standard for gathering data has been established in order to reduce the possibility of error caused by varying approaches to calculating the parameters influencing adsorption. The composition of the adsorbent contains either zero or a negligible amount of nitrogen. Nitrogen-doped carbon-based adsorbents demonstrate superior CO_2_ adsorption capacity and heightened adsorption selectivity compared to their non-nitrogen counterparts. This improvement can be attributed to the significant enhancement of base adsorption sites within these adsorbents due to the presence of nitrogen^66^. Therefore, to prevent errors, nitrogen-free sorbents were investigated. All specific surface areas were calculated using the BET equation from nitrogen adsorption at a temperature of 77 K. In this database, the total volume of the adsorbent was estimated from the absorption of liquid nitrogen at a relative pressure of 0.95–0.99. The Dubinin–Radushkevich (D–R) equation was used to obtain the volume of micropores. The volume of mesopores is calculated by subtracting the volume of micropores from the total volume of the adsorbent. The unit of BET surface area was square meters per gram (m^2^/g), the volume of micropores and mesopores was cubic centimeters per gram (cm^3^/g), the temperature was in degrees Celsius (C), the pressure was in bar, and the amount of carbon dioxide adsorption is based on millimoles of adsorbent per gram (mmol/g).

**Table 2:** The range of data employed in this study.

Author	SBET (m^2^/g)	Vmeso (cm^3^/g)	Vmicro (cm^3^/g)	T (C)	P (bar)	CO_2_ uptake (mmol/g)	References
Durá et al.	241–391	0.18–1.13	0.06–0.42	25	5–10	0.7–3.3	^45^
Hao et al.	618–887	0.075–1.115	0.217–0.347	25	0.04–1	0.35–2.6	^67^
Travis et al.	1624–2785	0.073–0.644	0.589–0.793	0–50	0.1–10	0.4–23.23	^68^
Lee and Park	2162–2318	0.075–0.207	0.917–0.990	25	0.1–1	0.68–5.56	^69^
Sevilla and Fuertes	970–2660	0.16–0.72	0.12–1.03	0–50	0.1–1	0.2–5.8	^66^
Sevilla and Fuertes	1260–2850	0.06–0.24	0.55–1.23	0–50	0.3–1	1.25–6.6	^70^
Wahby et al.	2445–3100	0–0/14	1.03–1.45	0–50	1	1.75–8.63	^71^
Hong et al.	1023–2750	0.011–0.423	0.403–1.042	0–50	0.2–1	0.4–6.05	^40^
Casco et al.	1770–3575	0.03–0.61	0.7–1.22	25	0.2–40	0.64–31.8	^49^
Ludwinowicz and Jaroniec	460–2130	0.04–0.32	0.2–0.78	0–120	0.15–1	0.2–6.7	^72^
Adeniran and Mokaya	1479–2925	0.16–0.32	0.36–1.18	0–25	5–20	8–25.5	^73^
Parshetti and Chowdhury	1080–2510	0.08–0.55	0.09–0.55	0–50	0.5–1	0.393–5.229	^74^
Estevez et al.	1477–2772	0.931–10.073	0.014–0.251	25	0.1–1	0.2–3.7	^75^
Srinivas et al.	1260–2734	0.435–4.919	0.139–0.611	25–50	2–29	1.6–21	^76^
Singh et al.	637–1122	0.092–0.192	0.25–0.5	0	2–30	4.5–15.5	^77^
Sevilla et al.	1270–1690	0.01–0.05	0.49–0.67	25	0.1–50	0.5–14.5	^43^
Hao et al.	670	0.26	0.2	0–25	0.1–1	0.68–3.3	^78^
Balahmar et al.	1202–2980	0.1–1.8	0.3–0.9	0–25	0.15–20	0.4–24	^79^
Hirst and Taylor et al.	1511–2610	0.11–0.41	0.54–0.74	25	0.15–20	0.9–19.7	^80^
Zhang et al.	55–1327	0.16–0.61	0–0.35	25	5	5.46–0.32	^44^
Zhang et al.	324–923	0.02–0.1	0.14–0.46	0–25	0.15–1	1.16–5.91	^81^
Choma et al.	218–3870	0.05–0.91	0.07–1.16	0–25	0.01–1.14	0.05–7.61	^82^
de Souza et al.	599–2710	0.2–0.89	0.13–0.65	25	1.01	1.38–4.37	^83^
Park et al.	310–885	0.015–0.156	0.109–0.327	40	0.01–1.01	0.16–3.65	^84^
Li et al.	1634–3337	0.204–1.512	0.535–1.286	0–25	1	2.9–6.4	^85^
Ma et al.	972–2305	0.1–0.279	0.393–0.934	0–25	0.15–1	2.99–0.87	^86^
Haffner-Staton et al.	1121–2487	0.04–0.21	0.45–1.01	25	0.15–20	0.8–20.3	^87^
Khodabakhshi et al.	681–2252	0–0.19	0.25–0.85	25	0.15–10	0.9–12.4	^88^
Coromina et al.	1048–3537	0.08–1.64	0.43–1.23	25	0.15–20	0.4–22.5	^89^

**Figure 1:**
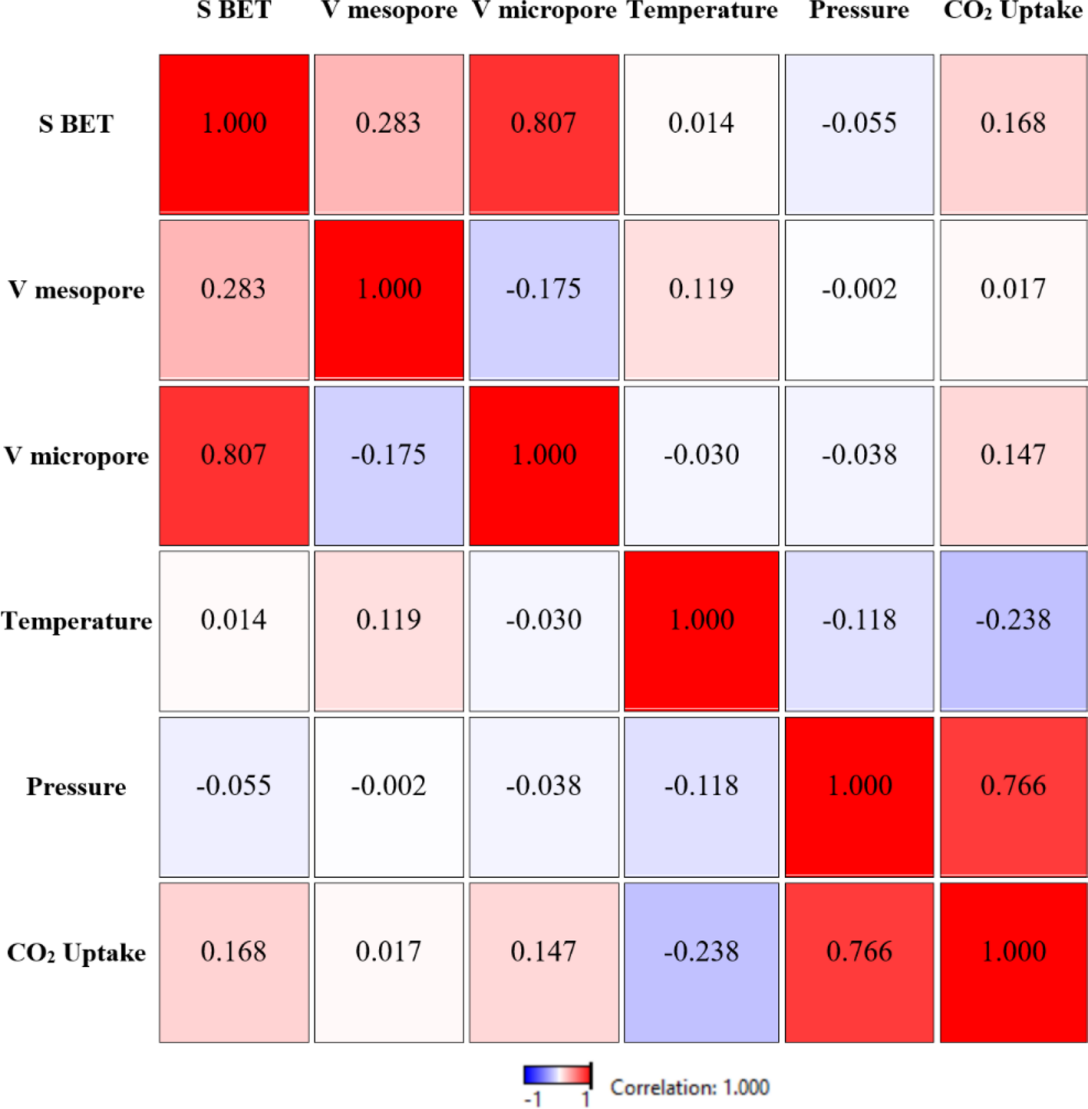
Pearson correlation matrix between any two variables of porous carbon adsorbents and CO_2_ adsorption capacity based on the total database.

The Pearson correlation coefficient matrix is the ratio of the covariance of each pair of adsorbent variables to the product of their standard deviations. Based on the Pearson correlation coefficient matrix (Fig. 1), there is no significant linear correlation between adsorbent textural properties and CO_2_ uptake capacity. The correlation between adsorption capacity and the volume of mesopores and micropores (R = 0.017 and R = 0.147 respectively) indicates a weak relationship. Moreover, there was a strong positive correlation (R = 0.807) between micropore volume and BET surface. However, CO_2_ adsorption capacity demonstrated a robust positive correlation with pressure (R = 0.776) and a moderately poor relationship with temperature (R = − 0.238).

In this study, 1345 data points were acquired, of which 1300 were used to develop the artificial neural network model. In addition, the 45 data were chosen randomly to predict carbon dioxide adsorption. MATLAB software arbitrarily separated 80% of the 1300 data points for training, 10% for validation, and another 10% for testing. Selecting the proper input and output is one of the important stages in creating a neural network model. According to what was stated in the introduction, previous research has demonstrated that in addition to operational parameters like temperature and pressure, the textural properties of the adsorbent, including the BET surface area, volume, especially the volume of mesopores, and the volume of micropores, significantly influence the adsorption process. As a result, the variables of BET surface, mesopore volume, micropore volume, temperature, and pressure are chosen as network input variables. The objective of creating a model is to predict the carbon dioxide adsorption capacity. Therefore, the amount of carbon dioxide adsorbed is deemed the network's output. To reduce the impact of parameters with greater magnitude on the ANN design, the entire database was standardized in the range of 0–1 (Supplementary Information).

## Theory and methodology

### Artificial neural networks

An artificial neural network (ANN) is a computational model that employs the architecture of the human brain to predict intricate and non-linear systems. Within the network's structure, artificial neurons are interconnected across the input, hidden, and output layers^90^. The neural network is exposed to input–output pairs and undergoes training to predict the output variables. The training process establishes the connection strengths among the processing neurons through the utilization of an appropriate training algorithm. The biases between the layers and the connectivity weights, thus, influence the input signals. An activation function is employed to modify the sum of these signals, aiming to minimize the disparity between the predicted output and the actual output data. The commonly utilized activation functions include purelin (Eq. 2), logsig (Eq. 3), and tansig (Eq. 4).

2 $$ {\text{f(x}}) = x $$

3 $$ {\text{f(x)}} = \frac{1}{{1 + e^{ - n} }} $$

4 $$ {\text{f(x) = }}\frac{2}{{1 + e^{ - 2n} }} - 1 $$

Prominent types of ANNs include the radial basis function (RBF) and the multilayer perceptron (MLP)^91^. It is crucial to highlight the key distinction between these networks, which lies in the functioning of neurons.

The RBF-ANN architecture consists of an input layer, a hidden layer, and an output layer. The neurons within the hidden layer utilize radial basis functions as their activation functions. Through the utilization of a linear optimization strategy and the adjustment of weights during the minimization of mean square error, this algorithm can ascertain the optimal solution.

As mentioned earlier, the multilayer perceptron (MLP) is an alternative form of ANN. This algorithm comprises multiple layers, with the input layer being the first and the output layer being the last. Intermediate and hidden layers connect the input and output layers, where various forms of activation functions can be applied^92^. Additional information about MLP and RBF algorithms can be found in the literature^93,94^.

### MLP training algorithms

The algorithm's learning process involves the forward propagation of data and the backward propagation of errors. Input data enters the model through the input layer without initial processing, undergoes initial processing in the hidden layer, and then gets to the output layer. If the difference between the network's predictions and the actual outputs does not meet the required level of accuracy, an error will be backpropagated through the network for further adjustment.

The backpropagation of errors works by propagating the difference between the network's output and the actual output back through the hidden layer to the input layer. The network's training procedure continues until the error between the network result and the actual output falls within the allowed tolerance or reaches a predetermined number of learning cycles^28^.

Furthermore, there are six distinct classes of backpropagation algorithms: adaptive momentum, self-adaptive learning rate, resilient backpropagation, conjugate gradient, Quasi-Newton, and Bayesian regularization^95^.

#### Adaptive momentum

The gradient descent (GD) algorithm is employed to determine an optimal set of internal variables for model optimization in machine learning and deep learning problems. Typically, gradient descent involves three steps: (1) initializing the internal variables, (2) evaluating the model using the internal variables and the loss function, and (3) updating the internal variables in a manner that moves toward optimal points. The gradient descent technique involves an iterative process as shown in Eq. (5).

5 $$ w^{i + 1} = w^{i} - \nabla f(w^{i} )\eta^{i} $$

In the given equation, w^i^ represents the set of variables that need to be updated, ∇f(w^i^) represents the gradient of the loss function f concerning the set of variables w^i^, and η denotes the learning rate. The value of η can be a constant or determined through a one-dimensional optimization along the training direction at each step. The primary objective of gradient descent is to locate the global minimum points that minimize or maximize the loss function, making this process essential in the optimization procedure for the loss function^96^.

Gradient descent with momentum backpropagation (GDM) is a training algorithm that utilizes batch steepest descent with an enhanced convergence rate. It incorporates momentum to adapt to trends in local gradients and error surfaces, thereby mitigating the risk of getting stuck in shallow local minimums. By employing momentum, GDM achieves faster convergence during the training process^97^.

#### Self-adaptive learning rate

The efficacy of the algorithm relies on the appropriate configuration of the learning rate. If the learning rate is set too high, it can result in instability, while setting it too low can lead to slow convergence. Determining the optimal learning rate prior to training is impractical, as it varies during the training process depending on the algorithm's progress across the performance surface.

To enhance the performance of the gradient descent algorithm, an adaptive learning rate can be utilized, allowing for adjustments during training. The primary objective of an adaptive learning rate is to maintain a maximal learning step size while ensuring stability in the learning process^98^.

The conventional steepest descent (GD) backpropagation algorithm employs a fixed learning rate parameter during the network's training process. Nevertheless, the algorithm's performance significantly relies on the specific value assigned to this learning rate parameter. To address this, the gradient descent with an adaptive learning rate backpropagation (GDA) algorithm was created, enabling an adaptive adjustment of the learning rate parameter. This adaptive strategy strives to optimize the magnitude of each learning step while maintaining the stability of the learning process. In the GDA algorithm, the optimal value for the learning rate parameter varies depending on the trajectory of the gradient on the error^97^. The training algorithm referred to as gradient descent with momentum and adaptive learning rate backpropagation (GDX) integrates both adaptive learning rate and momentum training principles. It is similar to the GDA algorithm, but with the addition of a momentum coefficient as a training parameter. Consequently, the weight vector is updated using a similar approach as in GDM, while incorporating a variable learning rate as seen in GDA.

#### Resilient backpropagation (RP)

Resilient backpropagation (RP) is typically applied to eliminate the negative consequences of partial derivative values. This algorithm has the advantage of being significantly quicker than the standard reduction algorithm^95^. In the hidden layers of multilayer networks, sigmoid activation functions are typically utilized to restrict the output range. As the input value increases, the slope of the Sigmoid functions tends to approach zero. This poses a challenge when training a multilayer network using gradient descent and Sigmoid functions, as the gradients can become exceedingly small, resulting in weight and bias updates that deviate significantly from the optimal values. The resilient backpropagation training algorithm aims to mitigate the adverse impact of small partial derivatives. In this approach, only the sign of the derivative is utilized to determine the weight update direction, while the derivative's actual value does not affect the weight update. The magnitude of the weight change is determined by a distinct update value^98^.

#### Conjugate gradient

The conjugate gradient algorithm, which combines elements of gradient descent and Newton's method, enhances the convergence rate of artificial neural networks by eliminating the requirement to measure, store, and invert the Hessian matrix. It explores conjugate directions in a coordinated manner, leading to faster convergence compared to the directions followed by gradient descent. The algorithm establishes the sequence of training directions using the equation provided below.

6 $$ y^{i + 1} = v^{i + 1} + y^{i} c^{i} $$

Utilizing the primary training direction vector

7 $$ y^{0} = - v^{0} $$

where y represents the training direction vector, c denotes the conjugate parameter, and i is set as the negation of the gradient in all scenarios^96^. The conjugate gradient algorithm's parameter improvement procedure is defined by

8 $$ w^{i + 1} = w^{i} + y^{i} \eta^{i} $$

where ${\upeta }^{{\text{i}}}$ is the learning rate, which is determined by line minimization normally.

The standard backpropagation algorithm modifies weights in the direction of the steepest descent, but this does not guarantee the quickest convergence. Conjugate gradient algorithms expedite convergence by exploring conjugate directions. The initial iterations involve performing the steepest descent, conducting line searches, and combining the direction with the previous search direction. The determination of the new search direction depends on a constant value, which is calculated in the Fletcher–Reeves update[conjugate gradient backpropagation with Fletcher–Reeves update (CGF) algorithm] as the difference between the squared norm of the current gradient and the squared norm of the previous gradient^99^. The constant employed to determine the updated search direction in the Polak–Ribiére update, as part of the conjugate gradient backpropagation with the Polak–Ribiére update (CGP) algorithm, is calculated as the inner product of the previous gradient change and the current gradient, divided by the squared norm of the previous gradient. In contrast to the Fletcher–Reeves method, which involves three vectors, the Polak–Ribiére update requires a marginally higher storage capacity^97^.

In every conjugate gradient algorithm, the search direction is regularly reset to the inverse of the gradient. While other reset techniques can enhance training effectiveness, the typical reset point occurs when the number of iterations matches the number of network parameters (weights and biases). The Powell–Beale restarts [conjugate gradient backpropagation with Powell–Beale restarts (CGB) algorithm] is an example of such a reset technique. Powell introduced a restart strategy for enhancing training effectiveness, building upon a suggestion from Bill. This strategy triggers a restart when there is limited orthogonality between the current gradient and the previous gradient. Unlike Polak–Ribiére, the Powell–Beale algorithm demands slightly greater storage capacity^97^.

The three previously discussed conjugate gradient techniques require a line search after each iteration, which can be computationally expensive due to the need to compute the network output for all training inputs multiple times. To address this issue and significantly reduce the number of calculations required per iteration, the scaled conjugate gradient backpropagation (SCG) training technique was developed. However, SCG may require more iterations than the other conjugate gradient algorithms to achieve convergence. The storage requirements of the SCG algorithm are comparable to those of the CGF algorithm. In the majority of problems, SCG yields a superlinear convergent. It is at least an order of magnitude quicker than the backpropagation algorithm in terms of performance. Using a mechanism for resizing the step size, SCG avoids a lengthy search by row for learning iterations, making the algorithm speedier than other recently suggested second-order algorithms^97^.

#### Quasi-Newton

Quasi-Newton methods, a subset of variable metric techniques, are employed to identify local extremum points of functions. These methods draw their inspiration from Newton's method, designed to pinpoint stationary points of a function where the gradient equals zero. Newton's method assumes that the function can be locally approximated as a quadratic function in the vicinity of the optimal point. To accomplish this, it relies on the utilization of both the first and second derivatives of the function. In cases involving higher dimensions, Newton's method extends its application by incorporating the gradient and the Hessian matrix, which encapsulates the second derivatives of the function, with the objective of function minimization^100^.

Newton's method presents an alternative to conjugate gradient methods, known for its rapid convergence and optimization capabilities. It involves the computation of the Hessian Matrix, which leads to faster convergence compared to conjugate gradient methods. However, calculating the Hessian matrix for feedforward neural networks is challenging and computationally expensive. The BFGS Quasi-Newton backpropagation (BFGS) algorithm is well-suited for smaller networks, although it requires more storage and computational resources due to its complexity and cost^95^.

The one step secant backpropagation (OSS) training technique strikes a balance between conjugate gradient algorithms and full quasi-Newton algorithms. It offers reduced storage and computational requirements per iteration by computing the Hessian matrix only once per epoch and retaining it throughout the iteration. This approach determines the new search direction without explicitly calculating the inverse matrix. However, it necessitates additional computational and storage resources per iteration compared to conjugate gradient methods^97,99^

##### Levenberg–Marquardt backpropagation (LM)

In many problems, the Levenberg–Marquardt (LM) algorithm outperforms standard gradient descent and many other conjugate gradient methods. LM is a combination of the local search features of Gauss–Newton and the error reduction consistency afforded by the gradient descent algorithm. The feedforward network training based on LM is considered an unconstrained optimization issue. The Levenberg–Marquardt algorithm was developed to approximate the training speed of second-order methods without explicitly computing the Hessian matrix. In the case where the performance function of feedforward networks can be expressed as a sum of squares, the Hessian matrix can be estimated using the following approximation^101^:

9 $$ H = J^{{\text{T}}} J $$

The calculation of the gradient can be expressed in the following manner:

10 $$ g = J^{T} e $$

In this context, the Jacobian matrix denoted as J comprises the first derivatives of network errors concerning weights and biases, while e represents the vector of network errors. The Jacobian matrix can be obtained using a standard back-propagation technique, which is notably less intricate than the computation of the Hessian matrix. The Levenberg–Marquardt algorithm utilizes this approximation of the Hessian matrix in the subsequent Newton-like update iteration^98^.

11 $$ x_{k + 1} = x_{k} - [J^{T} J + \mu I]^{ - 1} J^{T} e $$

When the value of scalar μ equals zero, it corresponds to employing an approximation of the Hessian matrix in Newton's method. This results in a gradient descent with shorter steps when μ is large. Newton's method exhibits faster and more accurate convergence near an error minimum; thus, the goal is to transition to Newton's method as early as possible. Accordingly, μ is decreased after each successful step (improvement in the performance function) and only increased when a tentative step leads to an increase in the performance function. The performance function consistently decreases with each iteration in the algorithm by following this approach^98^.

#### Bayesian regularization backpropagation (BR)

The conventional Backpropagation (BP) algorithm can encounter the problem of overfitting, which manifests as reduced bias and increased variance. Conversely, the Bayesian regularization of artificial neural networks (BRANN) exhibits superior generalization capabilities. The BRANN minimizes the objective function F, which combines the mean squared error function E_D_ and the weight attenuation function E_W_. It probabilistically determines the optimal weights and parameters of the objective function. The objective function of the BRANN can be represented as^102^:

12 $$ F = \beta E_{D} + \alpha E_{W} $$

13 $$ E_{D} = \frac{1}{N}\sum\limits_{i}^{N} {(y_{i} - t_{i} )^{2} } = \frac{1}{N}\sum\limits_{i}^{N} {e_{i}^{2} } $$

14 $$ E_{W} = \frac{1}{2}\sum\limits_{i}^{m} {w_{i}^{2} } $$

In the given equation, α and β are hyper-parameters utilized to control the distribution of other parameters. w represents the weights, while m denotes the number of these weights. D refers to the training set data, represented as (x_i_, t_i_), where i ranges from 1 to N, indicating the total number of input–output pairs in the training set. y_i_ represents the output value corresponding to the i-th input–output pair in the training set^102^. The ANN model should produce nearly identical error rates for training and test data. Regularization is a technique that forces a neural network to converge to a set of weights and biases with reduced values. This makes the network's response more consistent and reduces the likelihood of data overfitting.

### Development of optimal ANN structure

**Figure 2:**
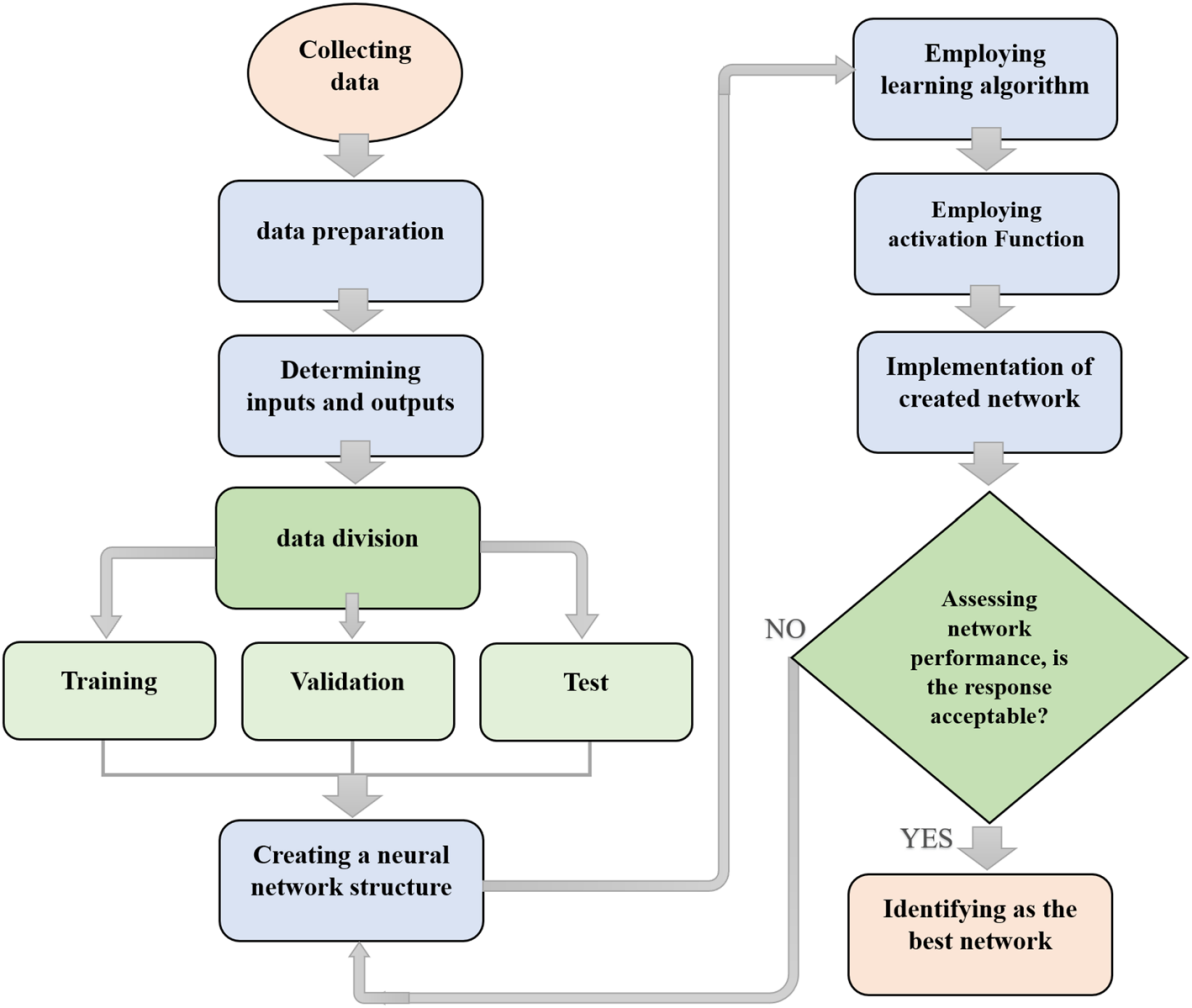
Schematic view of the MLP creation steps.

**Figure 3:**
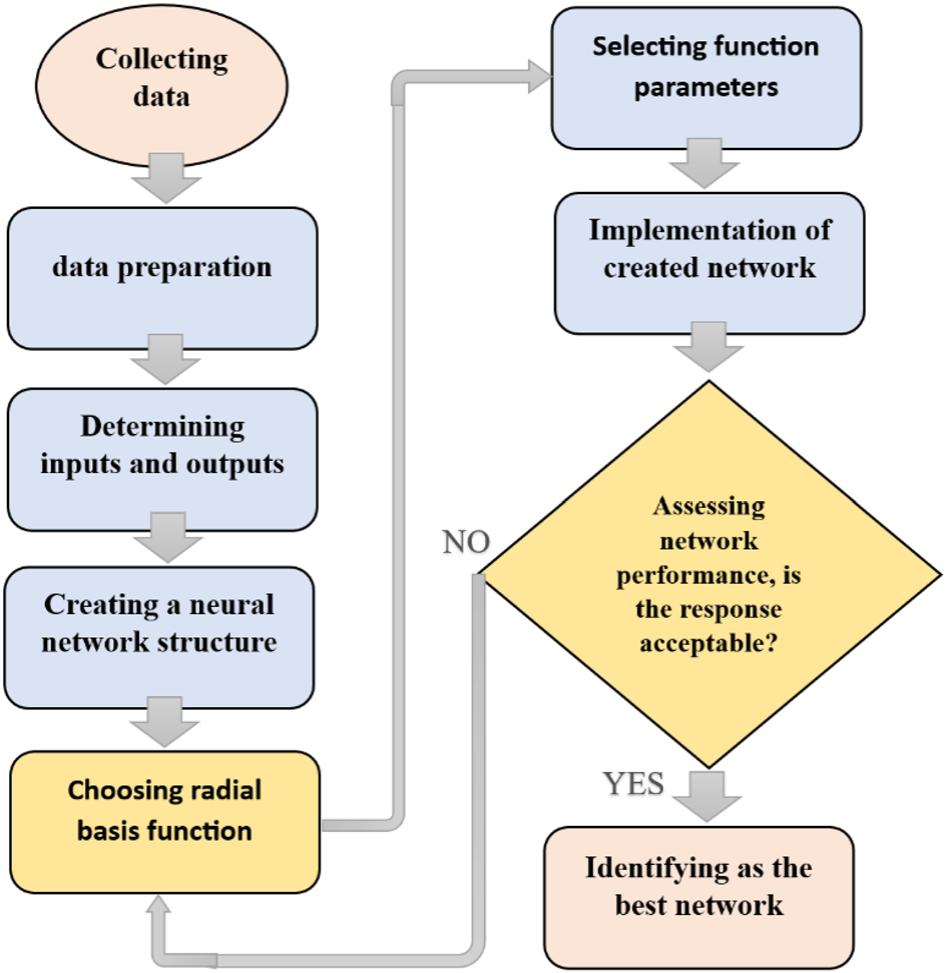
Schematic view of the RBF creation steps.

The process of constructing MLP and RBF neural networks is demonstrated in a step-by-step manner in Figs. 2 and 3, respectively. This process typically involves determining the network's architecture, training the network, and evaluating its performance. A trial-and-error methodology was employed to identify the optimal structure for the artificial neural network (ANN)^65^. The optimal ANN structure was subsequently determined based on the highest value of the low value of mean square error (MSE) (Eq. 15), Pearson's linear correlation coefficient (R) (Eq. 16), and the average absolute relative deviation percentage (AARD%) (Eq. 17)^103^.

15 $$ MSE = \frac{1}{N}\sum\limits_{i = 1}^{N} {(\alpha_{\exp } - \alpha_{cal} )}^{2} $$

16 $$ R = \frac{{\left[ {\sum\limits_{i = 1}^{N} {\left( {\alpha_{{\exp - \overline{{\alpha_{\exp } }} }} } \right)\left( {\alpha_{{cal - \overline{{\alpha_{cal} }} }} } \right)} } \right]}}{{\left[ {\sum\limits_{i = 1}^{N} {\left( {\alpha_{{\exp - \overline{{\alpha_{\exp } }} }} } \right)\sum\limits_{i = 1}^{N} {\left( {\alpha_{{cal - \overline{{\alpha_{cal} }} }} } \right)} } } \right]^{2} }} $$

17 $$ AARD\% = \frac{100}{N}\sum\nolimits_{i = 1}^{N} {\left| {\frac{{\alpha_{\exp } - \alpha_{cal} }}{{\alpha_{cal} }}} \right|} $$

Here α_exp_ represents the data points obtained from experimental measurements, while α_cal_ represents the corresponding data points calculated by the models. N denotes the total number of data points.

In prior studies^103,104^, researchers initially examined a predetermined network topology and explored several training algorithms on that specific network structure to identify the most optimum network configuration. After identifying the most effective learning algorithm, the researchers proceeded to determine the optimal network configuration by manipulating the number of neurons and layers inside the network. This would result in the loss of an essential part of the search space, namely the existence of a network with better performance but a different structure compared to the predetermined network structure for the trained network with a determined training algorithm. Notably, this approach highlights the importance of considering a broader range of network architectures to potentially discover superior configurations that may have been missed in the initial exploration.

In this study, the objective of the proposed method was to highlight the impact of the learning algorithm in determining the optimal network configuration. To achieve this, the development of optimal models for each training algorithm was initiated. Subsequently, an assessment was carried out to determine the most suitable network architecture and the optimal selection of activation functions for each model. This sequential approach enabled the systematic exploration of the impact of training algorithms on the Recognition of ideal network configurations within our specific domain of study. Thirteen different backpropagation training algorithms have been applied to train MLP neural networks. With the desire to determine the optimal neural network architecture for each training algorithm, two concealed layers of neurons ranging from zero to 50 are considered. Comparing four distinct combinations of logsig and tansig functions in the hidden layers of each algorithm's optimal architecture led to the detection of suitable activation functions. The initial assignment of weights and biases was randomly performed using MATLAB software. It is important to acknowledge that, to mitigate the impact of initial weight and bias assumptions on the outcomes, each MLP topology was executed at least three times, with only the most optimal result considered. This approach was employed to propose a model that exhibits enhanced and more precise performance, accounting for variations in the training process.

Similar to the MLP neural network, there are no specific rules for determining the optimal architecture of the RBF network. In the case of the RBF network, the number of hidden layers remains constant at one. The sole parameter of the network structure that requires determination is the number of neurons in the hidden layer. This parameter is established through a process of trial and error. In our research, we employ a Gaussian function with a radial basis activation function, and the spread value is dependent on the desired Gaussian function.

## Results and discussion

### Best MLP model

**Figure 4:**
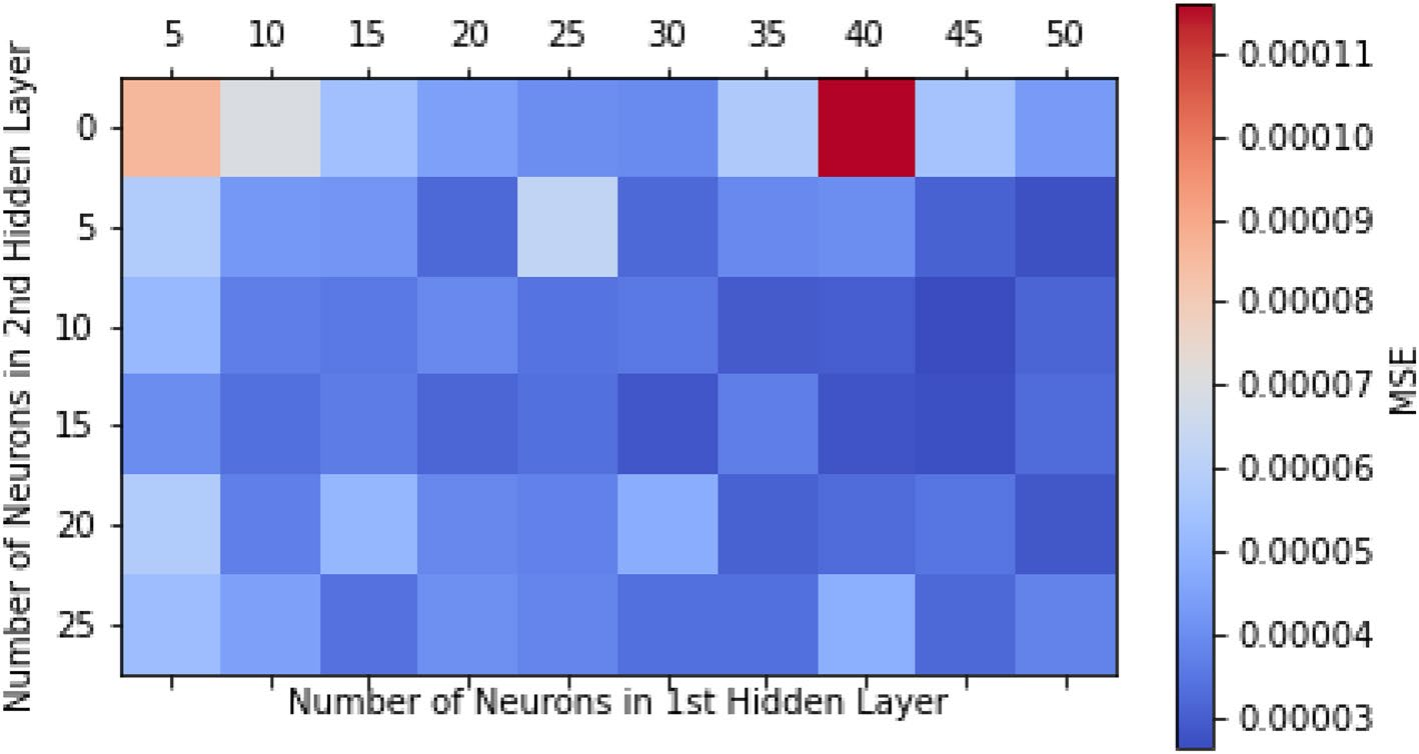
MSE for different neuron configurations.

Several structures of MLP were investigated for each training algorithm to determine the optimal ANN for predicting CO_2_ adsorption capacity. Figure 4 displays the best Mean Square Error (MSE) value achieved for each topology. The analysis of Fig. 4 suggests that a more complex neural network architecture with multiple hidden layers and specific neuron counts in those layers is necessary to achieve the best accuracy for the task at hand. This conclusion is drawn from the observed MSE values in the figure, which indicate that these configurations perform better than single-layer networks. To enhance the accuracy of models, for each network's training algorithm, the optimal ANN structure (determined by the lowest MSE) was utilized to evaluate four different combinations of activation functions. The outcomes of these evaluations are presented in Table 3. With the tansig activation function for the first layer and logsig for the second layer, the lowest MSE values for the BFG, RP, and CGB training algorithms are 9.01E−05, 4.78E−05, and 6.81E−05, respectively. The minimum MSE value of 9.23E−05 has been attained for the CGF training algorithm when the activation functions of the first and second layers are both logsig. While the activation functions of the first and second layers of other training algorithms are both tansig, the MSE has reached its minimum value. After obtaining the characteristics of the optimal networks for each training algorithm, these networks are applied to the prepared data and compared with each other. The outcomes of implementing networks with various algorithms and optimal architectures are listed in Table 4. For each of the applied networks, the MSE and correlation coefficient (R) for the training, validation, test, and total data are displayed.

**Table 3:** The outcomes of operating neural networks with various activation function combinations.

Training algorithm		Optimal structure	The MSE value for the first/second hidden layer activation function		
		logsig/logsig	logsig/tansig	tansig/logsig	tansig/tansig
LM	[5 45 10 1]	2.44E−05	3.13E−05	3.11E−05	3.15E−05
BR	[5 30 15 1]	2.72E−05	4.56E−05	5.23E−05	7.61E−05
BFG	[5 45 15 1]	1.12E−04	9.01E−05	2.59E−04	2.11E−04
RP	[5 50 15 1]	4.95E−05	4.78E−05	1.03E−04	8.09E−05
SCG	[5 50 5 1]	7.05E−05	1.34E−04	1.04E−04	2.97E−04
CGB	[5 40 10 1]	1.32E−04	6.81E−05	1.51E−04	8.13E−05
CGF	[5 45 10 1]	1.04E−04	1.19E−04	2.98E−04	9.23E−05
CGP	[5 40 10 1]	1.10E−04	1.17E−04	1.74E−04	7.54E−04
OSS	[5 50 25 1]	1.01E−04	2.08E−04	2.37E−04	2.20E−04
GDX	[5 45 15 1]	2.17E−04	2.25E−04	4.50E−04	4.20E−03
GDA	[5 50 5 1]	3.10E−04	3.35E−04	5.66E−04	6.91E−04
GDM	[5 30 5 1]	1.30E−03	2.20E−03	2.00E−03	2.50E−03
GD	[5 50 25 1]	5.61E−04	1.20E−03	8.30E−04	1.60E−03

**Table 4:** The results of implementing networks with diverse algorithms and optimal architectures.

Algorithm	Training		Validation		Test		Overall		Time (s)	Epoch
	MSE	R	MSE	R	MSE	R	MSE	R		
LM	1.9098E−5	0.9961	3.6342E−5	0.9944	7.3798E−5	0.9914	2.6293E−5	0.9951	5.4340	82
BR	2.4109E−5	0.9956	4.7848E−5	0.9886	3.0957E−5	0.9947	2.7167E−5	0.9949	8.8460	114
BFG	8.6843E−5	0.9836	1.1447E−4	0.9779	9.1297E−5	0.9842	9.0051E−5	0.9831	56.6110	146
RP	4.2602E−5	0.9918	6.2932E−5	0.9886	7.4511E−5	0.9884	4.7826E−5	0.9910	1.3630	322
SCG	6.7999E−5	0.9878	7.7137E−5	0.9779	8.3973E−5	0.9831	7.0510E−5	0.9867	0.7640	162
CGB	6.5655E−5	0.9878	8.2819E−5	0.9837	7.2497E−5	0.9869	6.8056E−5	0.9872	1.1940	130
CGF	9.2499E−5	0.9813	9.8389E−5	0.9848	8.4970E−5	0.9883	9.2335E−5	0.9827	2.2410	179
CGP	1.0577E−4	0.9803	1.3640E−4	0.9726	1.1196E−4	0.9772	1.1021E−4	0.9792	0.9580	115
OSS	8.9471E−5	0.9823	1.4326E−4	0.9790	1.4993E−4	0.9744	1.0090E−4	0.9810	2.1050	157
GDX	1.6878E−4	0.9678	1.9787E−4	0.9610	2.0777E−4	0.9665	1.7559E−4	0.9668	3.0440	1048
GDA	3.0120E−4	0.9474	3.5299E−4	0.9013	3.4069E−4	0.9297	3.1033E−4	0.9422	1.0010	373
GDM	4.4845E−4	0.9157	4.6946E−4	0.8906	3.6222E−4	0.9204	4.4192E−4	0.9138	10.8050	5000
GD	1.0873E−4	0.9784	1.7764E−4	0.9740	1.6627E−4	0.9712	1.2137E−4	0.9771	17.3030	5000

The LM training algorithm exhibits the highest level of accuracy among the considered training algorithms, showcasing an impressive MSE (mean squared error) of 2.62932E−05 across all datasets. This high accuracy extends to the training dataset with an MSE of 1.9098E−05, the validation dataset with 3.6342E−05, and even the test dataset with 7.3798E−05. In stark contrast, the GDM (gradient descent with momentum) algorithm performs less accurately, registering the lowest accuracy levels among the algorithms under examination. The superior performance of the LM algorithm in terms of accuracy can be attributed to its adaptability, use of second-order information, and efficient optimization in complex landscapes. Conversely, the GDM algorithm's lower accuracy may result from its reliance on fixed learning rates, sensitivity to initialization, and a greater tendency to get stuck in local minima.

The SCG algorithm, with a remarkably short runtime of 0.7640 s, stands out as the most time-efficient method for training neural networks in this study. In contrast, the BFG algorithm exhibits the longest training time, consuming 56.6110 s to complete the network training process. This substantial difference in runtime highlights the significant disparity in computational efficiency between these two optimization algorithms. the difference in training time between SCG and BFG likely arises from a combination of algorithmic differences, problem-specific factors, and the chosen settings or hyperparameters.

The LM training algorithm demonstrates efficient convergence within a relatively small number of epochs, specifically, 82 epochs. In contrast, both the GDM and GD training algorithms have reached the predefined maximum number of epochs, set at 5000 epochs, without achieving the desired convergence. This disparity in the number of epochs required for convergence underscores the distinct convergence behaviors of these algorithms. The LM algorithm's ability to achieve convergence within a limited number of epochs suggests its effectiveness in optimizing neural networks, while the protracted training process observed in GDM and GD may indicate challenges in navigating the optimization landscape.

**Figure 5:**
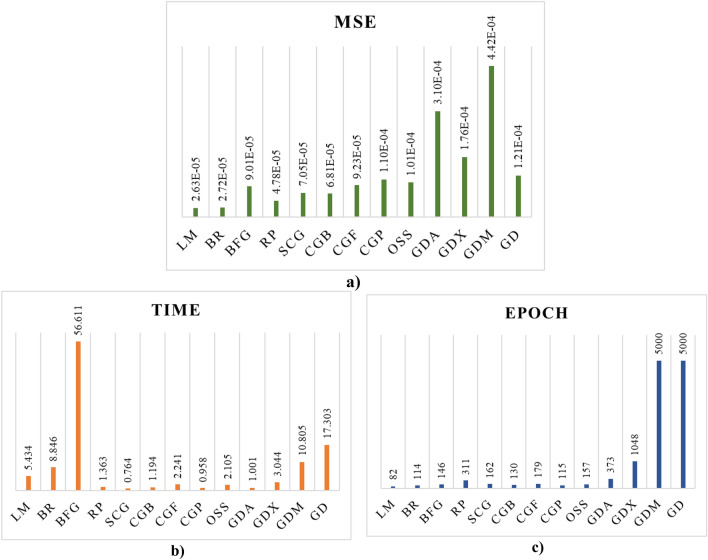
Comparing the efficacy of neural networks trained with various training algorithms with respect to: (**a**) accuracy, (**b**) run time, (**c**) number of epochs.

Figure 5 presents a comprehensive comparison of neural networks trained with various training algorithms, considering performance accuracy, run time, and the number of epochs. This comparison provides valuable insights into the trade-offs between these critical aspects of algorithm performance.

**Figure 6:**
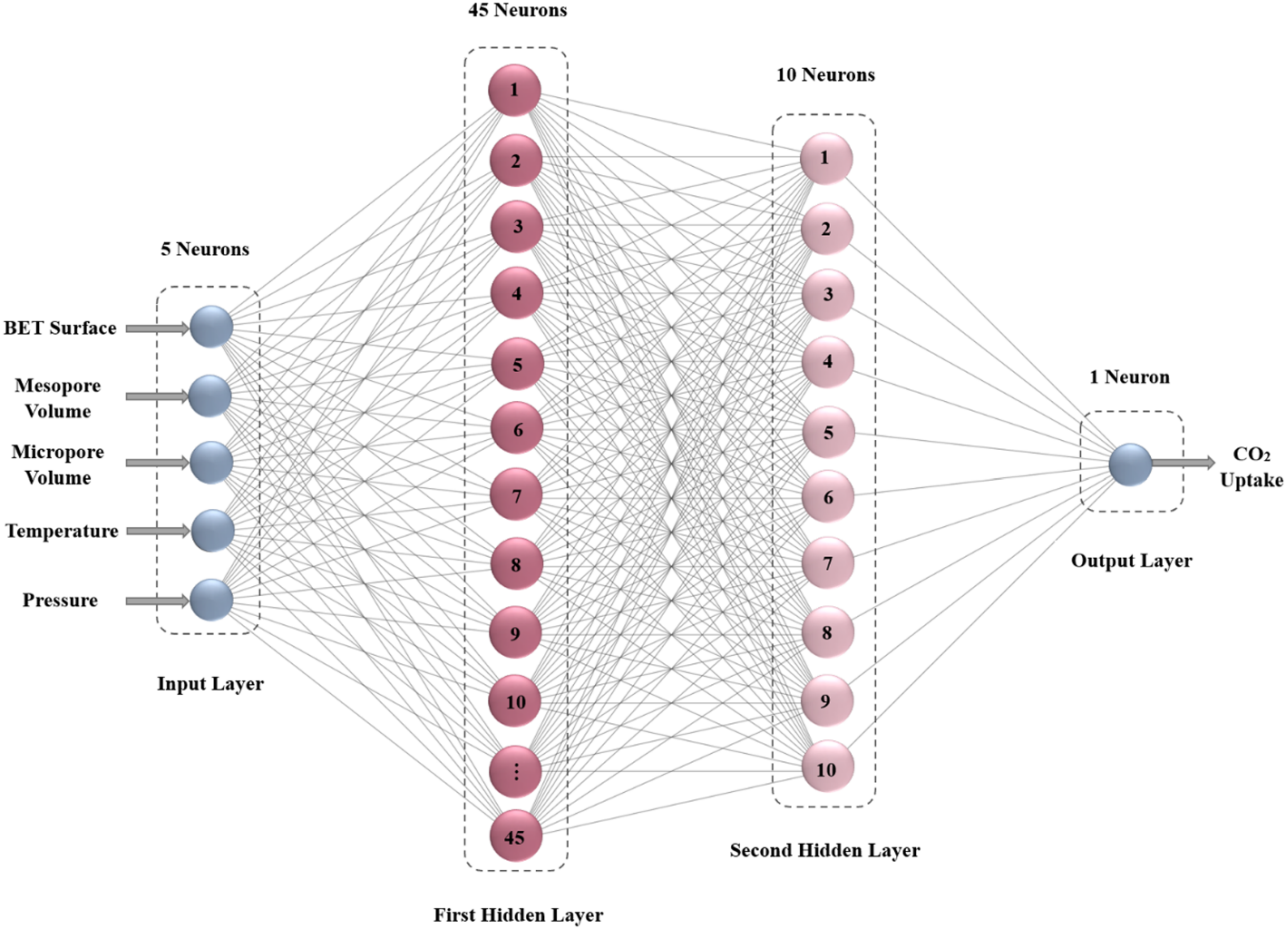
The structure of the optimal MLP network (trained with the LM algorithm).

As previously stated, the ANN trained with the LM backpropagation algorithm was chosen as the most effective training method due to its low mean square error (MSE < 2.6293E−05) and high correlation coefficient (R > 0.9951). Therefore, it is chosen as the optimal training algorithm to build the MLP neural network model for simulating and predicting carbon dioxide adsorption. Figure 6 depicts the structure of the optimal MLP network obtained.

**Figure 7:**
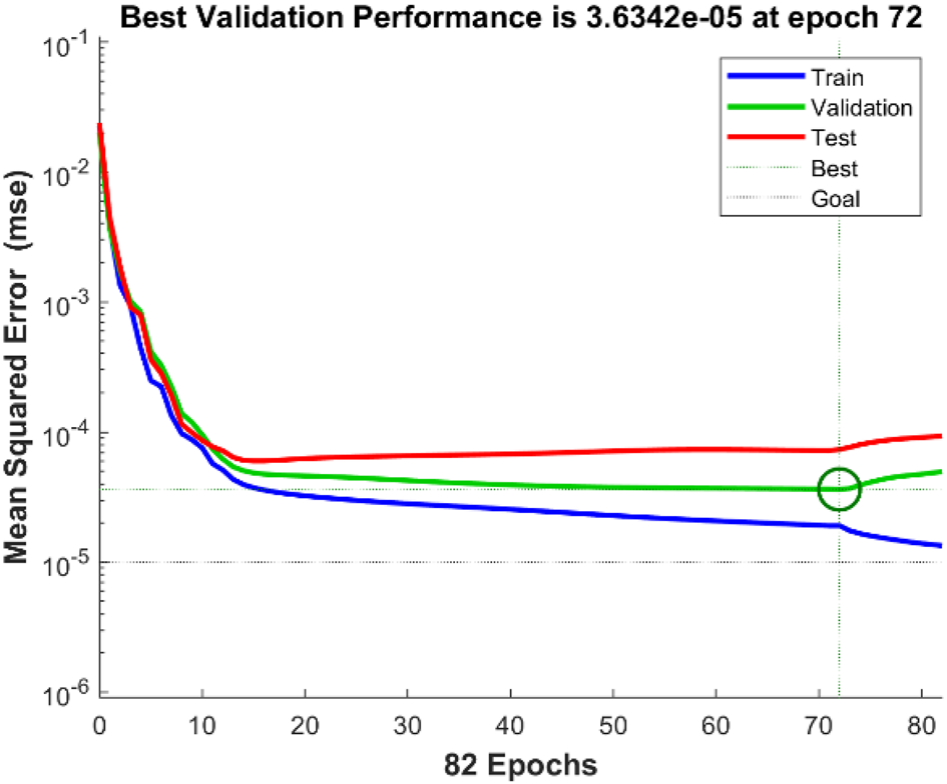
MSE by number of epochs for data sets n MLP network.

**Figure 8:**
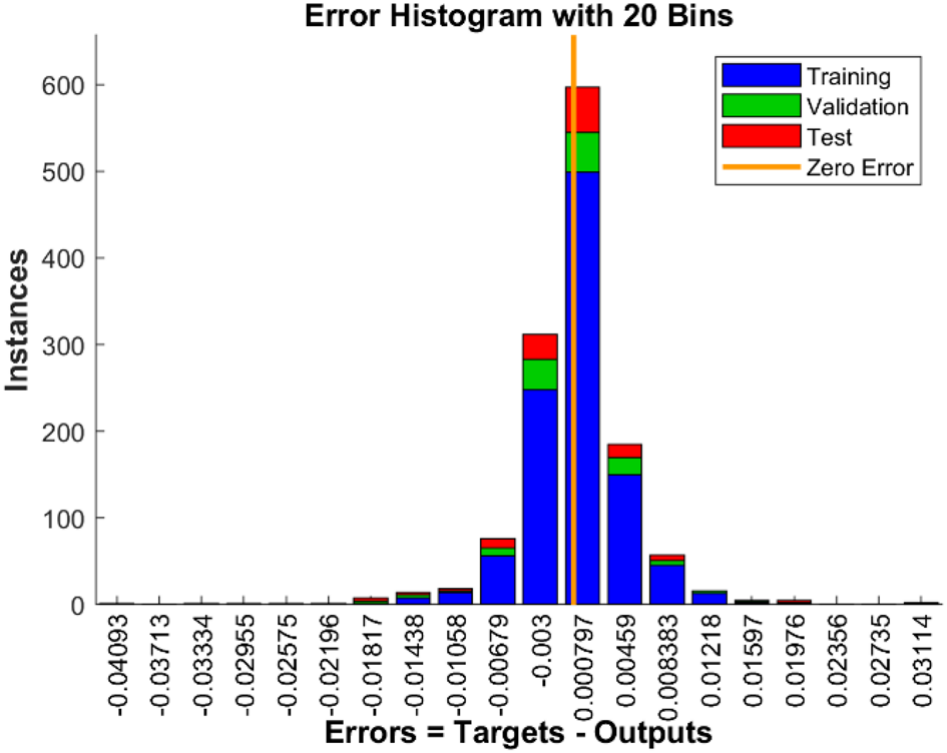
Error histogram plot for MLP network data sets.

**Figure 9:**
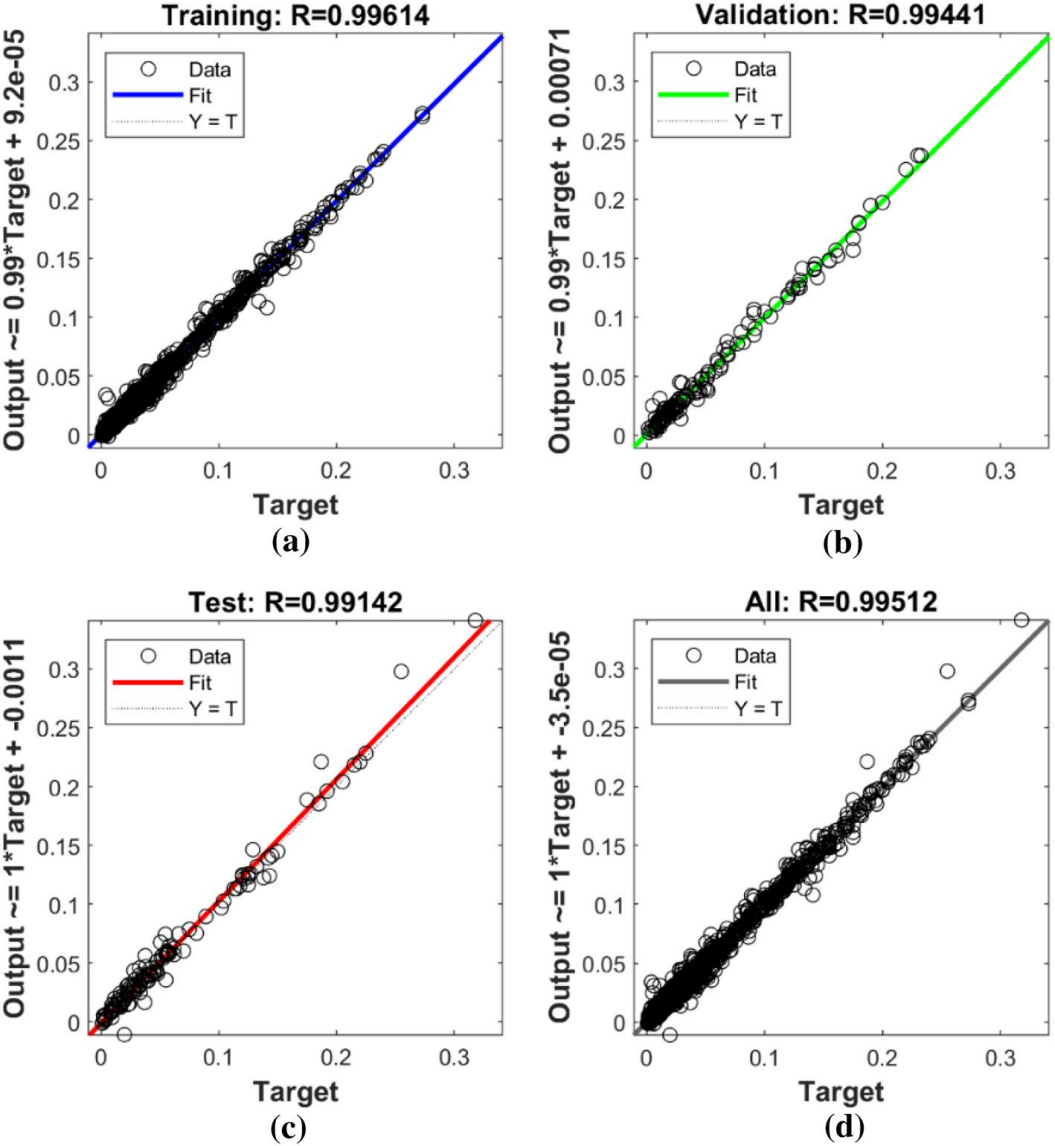
Comparing the experimental data with the results of the MLP neural network model with the (**a**) training, (**b**) validation, (**c**) test, (**d**) total data.

Figure 7 depicts the variation in mean square error as a function of the number of data application steps, where the optimal MLP model displays the best validation performance (3.6342E−05) at 72 epochs. In addition, the error histogram illustrates the operation of the neural network in Fig. 8. By comparing the collected experimental data with the data modeled by the MLP neural network in Fig. 9, it is evident that the experimental data and predicted data are highly congruent. In addition, experimental values are always associated with some error, necessitating the use of data with less error for a more accurate network. However, the R correlation coefficients for network training, validation, test, and total, were obtained as 0.99614, 0.99441, 0.99142, and 0.99512, emphasizing the reliability and accuracy of the chosen neural network model. This demonstrates the consistency model's performance across different data subsets and reinforces the robustness of the findings in this study. Therefore, neural networks are appropriate for modeling the CO_2_ adsorption on carbon-based adsorbents.

### Best RBF model

**Figure 10:**
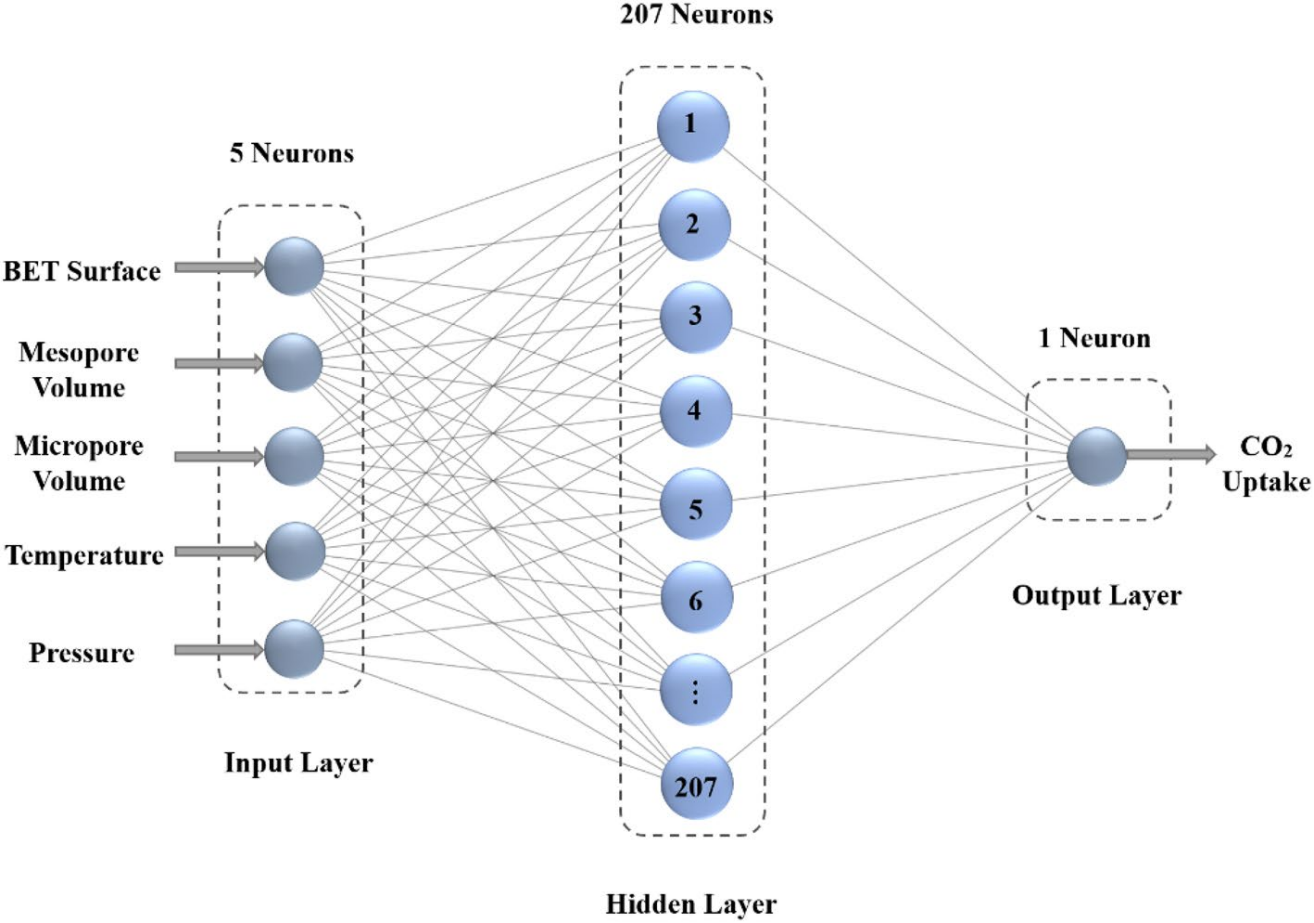
The structure of the optimal RBF network.

**Figure 11:**
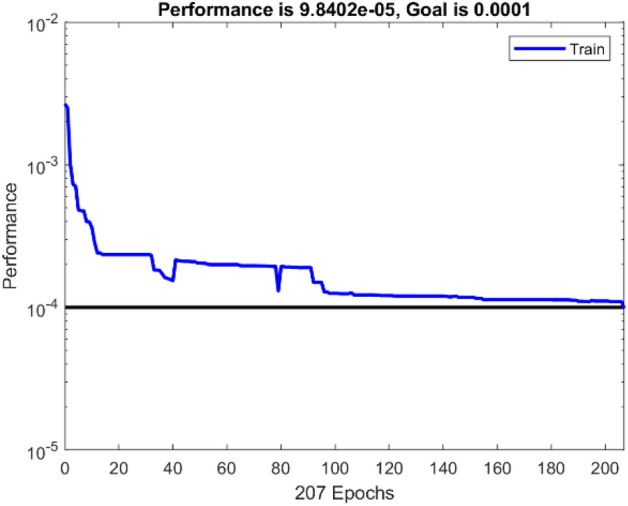
Variations in the MSE value of the RBF neural network based on the number of epochs.

**Figure 12:**
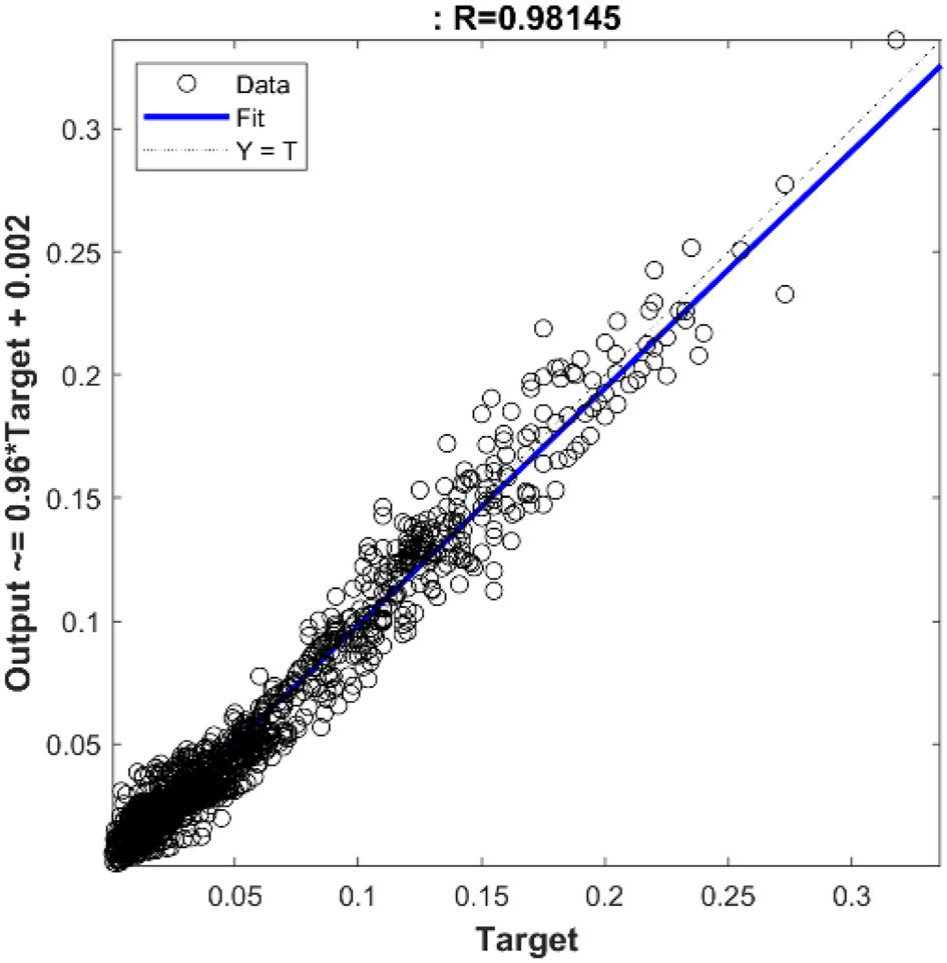
Linear regression between experimental data and RBF outputs.

As previously emphasized, it is essential to ascertain the optimal value for the spread parameter within the radial basis function for the RBF neural network. As evidenced by the data presented in Table 5, it becomes apparent that a spread value of 10 corresponds to the lowest observed mean squared error (MSE). The hidden layer encompasses a notable 302 neurons, signifying a relatively large quantity compared to other network configurations. However, it is noteworthy that this larger neuron count does not yield a substantially different mean squared error (MSE) value. Conversely, in the network with a spread parameter set at 9, a slight increment in MSE is observed. Nevertheless, this configuration is accompanied by a reduced number of neurons in the hidden layer, totaling 207. This reduction not only leads to diminished computational time but also translates into lower computational costs. Furthermore, it is worth noting that this particular network does not feature an excessively large or excessively small number of neurons within its hidden layer in comparison to alternative configurations. Additionally, its performance accuracy is notably high, making it the preferred choice as the optimal model for the RBF network. This selection is visually represented in Fig. 10, where the chosen RBF network configuration is depicted. The change in mean square error is displayed in Fig. 11, in which the optimal RBF model with 207 neurons in the hidden layer exhibits the best performance (9.8402E−05). In addition, a moderately good agreement between the RBF output values and the experimental data is observable in the regression diagram of Fig. 12, with the value of R equal to 0.98145.

**Table 5:** The MSE values for the spread range of 3 to 12 in the RBF network.

Spread	Neurons in hidden layer	MSE	Spread	Neurons in hidden layer	MSE
3	134	9.9689e−5	8	288	9.6546e−5
4	196	9.9356e−5	9	207	9.8401e−5
5	155	9.9423e−5	10	302	9.3096e−5
6	145	9.9570e−5	11	228	9.8682e−5
7	164	9.9495e−5	12	210	9.9267e−5

### Prediction of CO_2_ adsorption with new data

**Figure 13:**
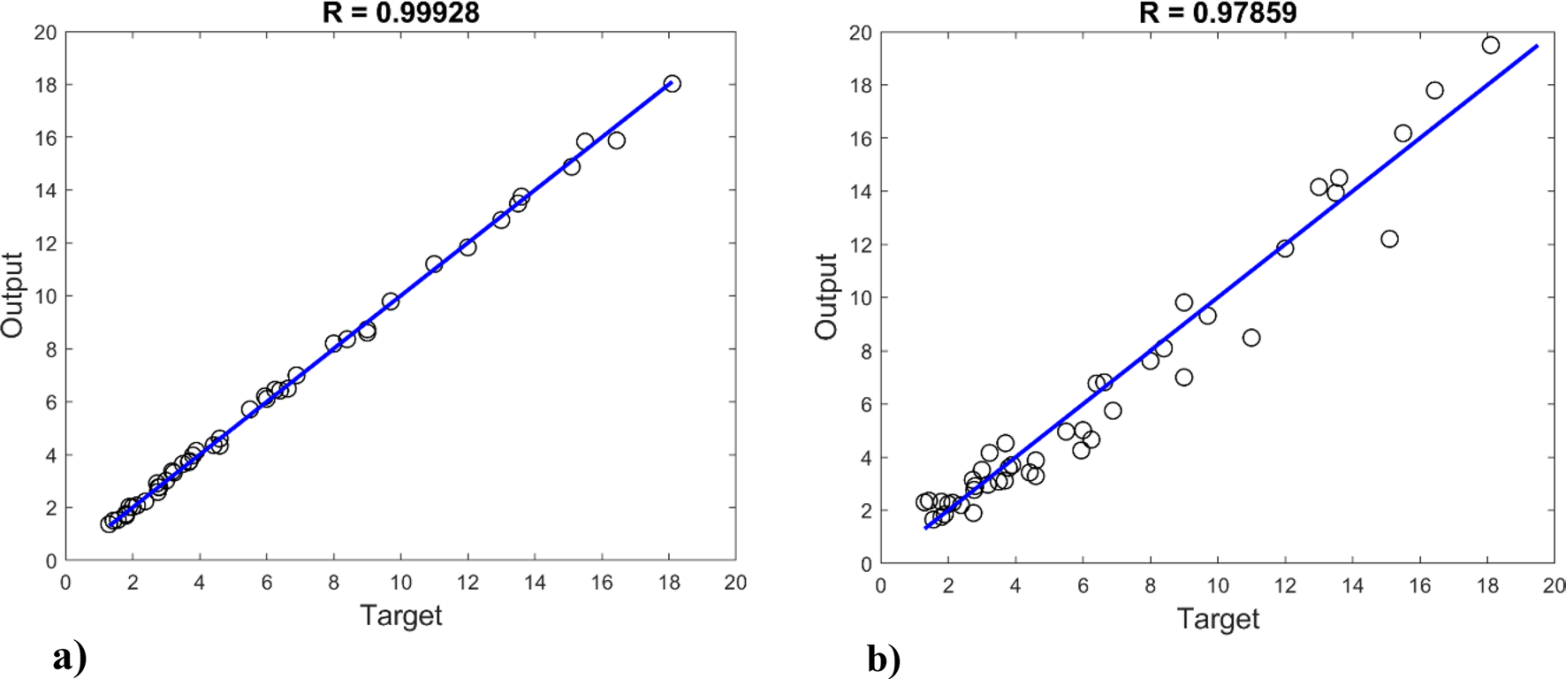
Linear regression between new experimental data and (**a**) MLP outputs, (**b**) RBF outputs.

In order to evaluate the efficacy of the created neural network models, the obtained MLP and RBF models are performed with 45 new data (which were initially separated from the data set), and the predicted CO_2_ adsorbed is compared to the experimental values. The results of CO_2_ adsorption prediction by MLP neural network models with various training algorithms are displayed in Table 6. The LM algorithm demonstrates the highest accuracy among all models, evidenced by the lowest AARD% value of 2.80 and the highest correlation coefficient of 0.9993. Additionally, the BR algorithm yields commendable results, with an AARD% of 4.27 and a correlation coefficient of 0.9988. The outcomes of RBF neural network models with varying spread values in predicting the quantity of CO_2_ adsorption are presented in Table 7. It is evident that the model achieving the lowest AARD% value, standing at 13.41% and associated with a dispersion of 9, attains the highest level of accuracy among all the models. For visual representation, Fig. 13 illustrates the linear regression between the predicted values for CO_2_ adsorption and the neural network outputs, considering both MLP and RBF models, using new data.

**Table 6:** Prediction of CO_2_ uptake by MLP neural network models with distinct training algorithms.

Algorithm	MSE	R	AARD%
LM	3.1753e−6	0.9993	2.80
BR	6.2280e−6	0.9988	4.27
BFG	8.1208e−5	0.9817	14.62
RP	3.2644e−5	0.9923	9.91
SCG	4.8543e−5	0.9889	13.01
CGB	4.2562e−5	0.9904	11.64
CGF	8.8721e−5	0.9804	15.14
CGP	9.3928e−5	0.9797	16.74
OSS	9.4982e−5	0.9785	16.83
GDX	1.4578e−4	0.9688	19.68
GDA	2.611e−04	0.9444	27.79
GDM	3.9733e−4	0.9095	31.79
GD	1.2558e−4	0.9719	17.85

**Table 7:** Prediction of CO_2_ uptake by RBF neural network models with distinct training algorithms.

Spread	MSE	R	AARD%
7	9.8275e−5	0.9784	13.97
8	1.1249e−4	0.9770	15.31
9	9.6342e−5	0.9786	13.41
10	9.3138e−5	0.9799	14.82

### Comparing MLP and RBF

Modeling and simulation of carbon dioxide adsorption on carbon base adsorbents by neural networks revealed that the MLP network (with the LM training algorithm) complies with experimental values more closely than RBF. Table 8 provides the MSE value and correlation coefficient derived from simulation and prediction with new data for both networks. The MLP deep neural network is more appropriate for modeling and simulating this process than the RBF network due to its higher correlation coefficient and lower mean square error values. As previously stated, the relation of Durá et al.^45^ is presented to predict the quantity of carbon dioxide adsorbed by micropore and mesopore volume. This model predicts the amount of carbon dioxide adsorbed on 12 distinct adsorbers using a square correlation coefficient of 0.9829. With a correlation coefficient of 0.9951 for more than 200 adsorbers at varying temperatures and pressures, it is evident that the MLP deep network model obtained through this study is more accurate and efficient.

**Table 8:** Comparing the performance of various models for the CO_2_ adsorption process.

Model	MSE	R
MLP	9.6293e-5	0.9951
RBF	9.8401e-5	0.9814
Durá et al.	Not mentioned	0.9829

### Evaluation of adsorption factors

**Figure 14:**
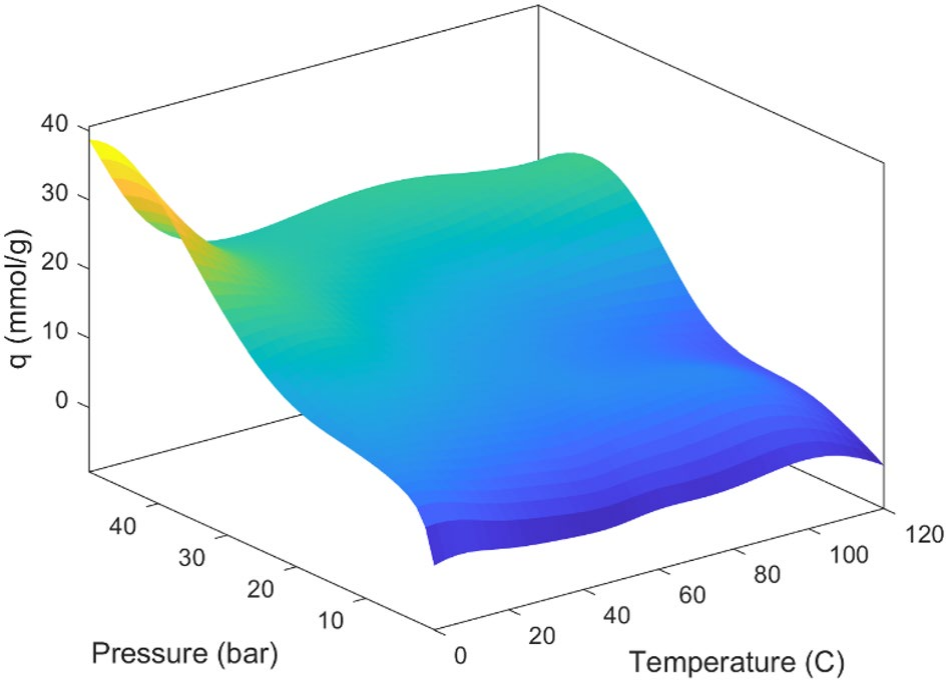
CO_2_ adsorption based on pressure and temperature for MLP trained with the LM training algorithm.

Figure 14 exhibits a three-dimensional graphical representation depicting the relationship between carbon dioxide adsorption, temperature, and pressure. This depiction assumes that the volumes of mesopores, micropores, and the BET surface remain constant, set at values of 0.75, 0.53, and 1510, respectively. The graph illustrates a notable upward trend in adsorption with increasing pressure, aligning with findings observed in pertinent studies^24,105^. Conversely, with an increase in temperature to 120 °C, a slight reduction in the adsorption becomes apparent. This decrease can be attributed to the exothermic nature of the adsorption process, whereby the concentration of adsorbed gas on the adsorber's surface diminishes as temperature levels rise^24,40^. According to the data presented in Fig. 14, the highest levels of CO_2_ adsorption are observed within the pressure range of 30–50 and the temperature range of 0–20.

**Figure 15:**
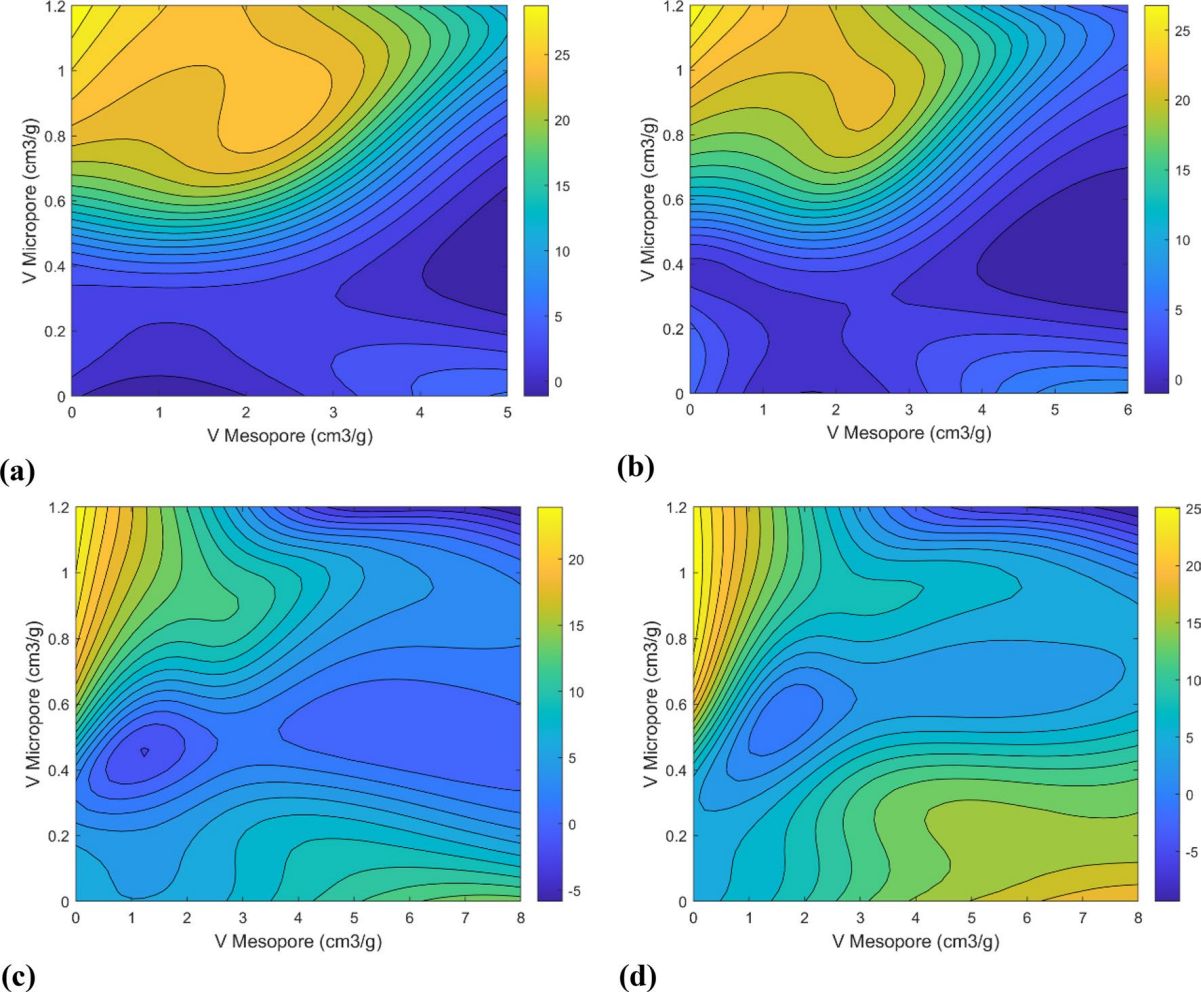
CO_2_ adsorption contours based on mesopore and micropore volume for MLP deep network with LM training algorithm at pressures (**a**) 1 bar, (**b**) 5 bar, (**c**) 15 bar, (**d**) 20 bar.

The carbon dioxide adsorption characteristics at 25 °C, a BET surface area of 500 square meters per gram, and pressures of 1, 5, 15, and 20 bar are presented in Fig. 15, with a focus on the role of mesopores and micropores. At 1 bar pressure, the influence of micropore volume in the range of 0.6–1.2 cm^3^/g on carbon dioxide adsorption is predominantly observed in Fig. 15a. This trend is sustained up to 5 bar, where a significant role for micropores is depicted in Fig. 15b. However, as pressures increase to 15 and 20 bars, the prominence of mesopore volume becomes more evident, as observed in Fig. 15c,d. Particularly at 20 bar, substantial growth in the quantity of adsorption within the mesopore volume range of 4–8 cm^3^/g, is exhibited. These findings are aligned with prior research^37,40,42,43^, which suggests that carbon dioxide adsorption is primarily governed by micropore volume at lower pressures and mesopore volume at higher pressures. This shift may be attributed to the saturation of micropores at higher pressures, necessitating the contribution of mesopores to achieve higher CO_2_ uptake^43^.

**Figure 16:**
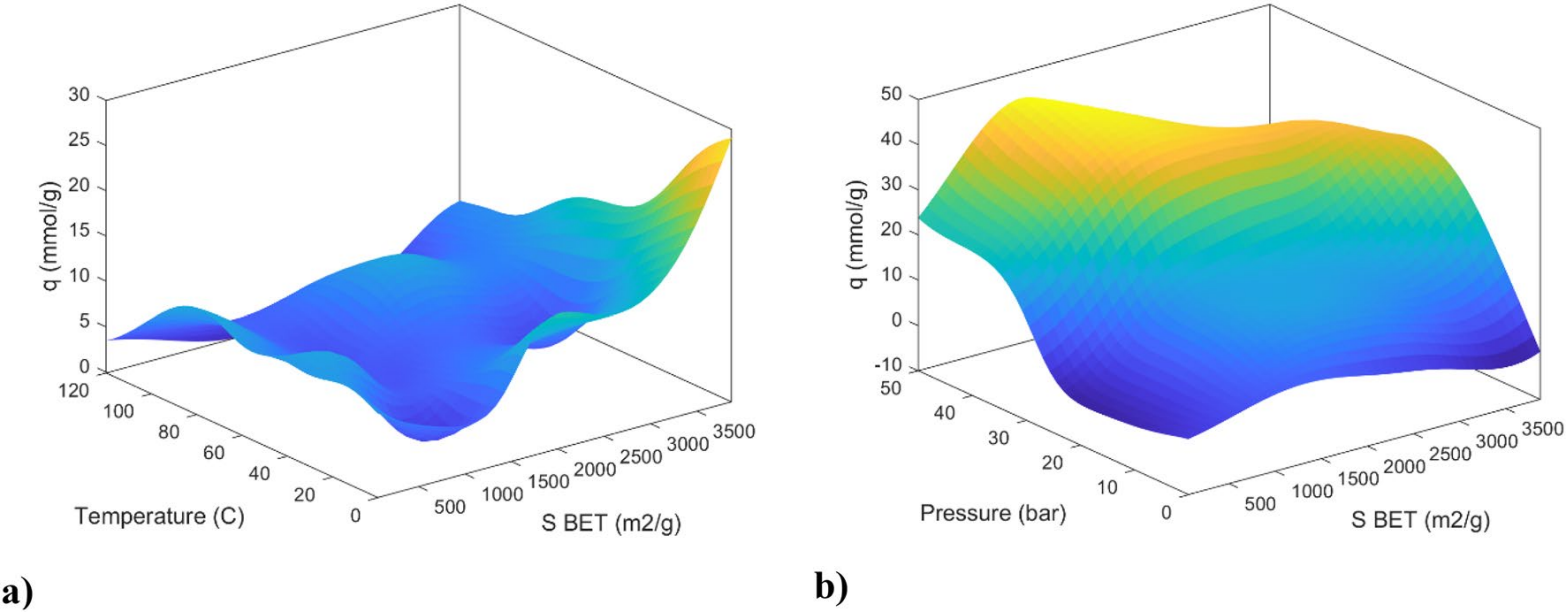
3D plots of CO_2_ adsorption based on (**a**) temperature and BET surface, (**b**) pressure and BET surface for MLP trained with the LM training algorithm.

Figure 16 presents the depiction of carbon dioxide adsorption onto the adsorbent, examining its dependency on BET surface area, temperature, and pressure. Figure 16a elucidates the outcomes under a fixed pressure condition of 5 bar while concurrently noting micropore and mesopore volumes of 0.53 and 0.75, respectively. Within this parameter range, the study observed that the maximum CO_2_ adsorption occurred at lower temperatures ranging from 0 to 60 °C, along with a higher BET surface area ranging from 2000 to over 3500 m^3^/g. Generally, a diminishing trend in carbon dioxide adsorption was noted as temperature increased. Conversely, as the BET surface area approached 2000, a notable increase in adsorption was recorded, followed by a modest decline, although it remained elevated.

In Fig. 16b, the findings are presented at a consistent temperature of 25 °Celsius, with micropore and mesopore volumes set at 0.75 and 4.5, respectively. Within this context, the study identified the peak adsorption occurring within a specified pressure range of 30–50 bars, in conjunction with a BET surface area ranging from 1000 to 2500 m^3^/g. Overall, it was observed that carbon dioxide adsorption exhibited a positive correlation with both increasing pressure and BET surface area. Nevertheless, it is important to note that the observed increase in surface area did not consistently result in a simultaneous increase in adsorption across all pressure levels. This observation suggests that a substantial specific surface area indeed enhances the adsorption capacity of CO_2_ but within a specific range of CO_2_ pressures^37^.

**Figure 17:**
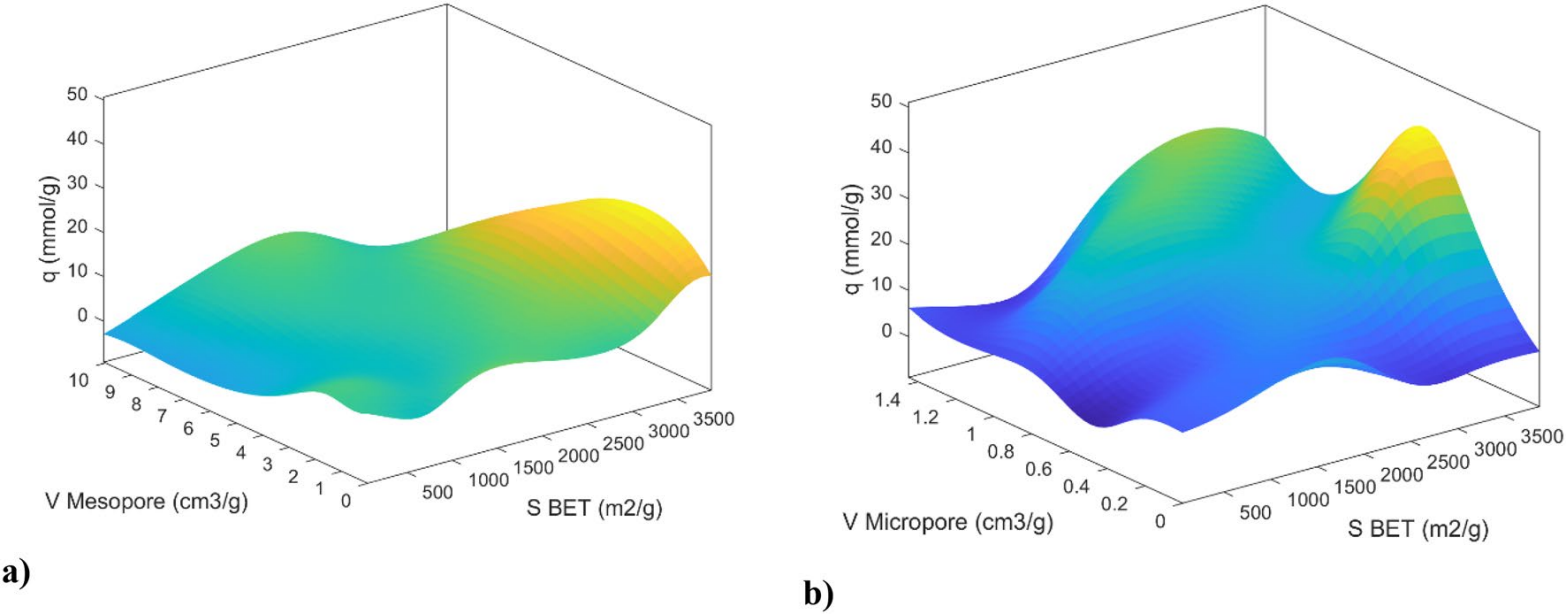
3D plots of CO_2_ adsorption based on (**a**) mesopore volume and BET surface, (**b**) micropore volume and BET surface for MLP trained with the LM training algorithm.

Figure 17 delineates the influence of BET surface area, mesopore volume, and micropore volume on CO_2_ adsorption. Within Fig. 17a, a discernible trend emerges, wherein carbon dioxide adsorption exhibits an ascending pattern in response to elevated BET surface area and mesopore volume values. This behavior is observed under specific conditions, including a micropore volume of 0.45 cm^3^/g, a temperature of 25 °C, and a pressure of 5 bar. Notably, the zenith of carbon dioxide adsorption manifests within a designated range, observed within 1 to 7 cm^3^/g for mesopore volume and 2500–3000 m^2^/g for BET surface area.

In Fig. 17b, conducted at 25 °C and 15 bar, with a mesopore volume of cm^3^/g, the paramount point of carbon dioxide adsorption is situated within the domain defined by micropore volume values ranging from 0.4 to 0.8 and BET surface area values spanning from 3000 to 3700 m^2^/g. Furthermore, a substantial quantity of adsorption is discernible within the span characterized by micropore volume from 1 to 1.4 and BET surface area from 1500 to 3500 m^2^/g. These findings signify the distinct influence exerted by micropore volume at lower BET surface area values and the accentuated impact of BET surface area at reduced micropore volumes.

It is imperative to underscore the formidable challenge posed by the synthesis of porous carbon materials concurrently possessing high BET surface areas (indicative of substantial micropore volume) and low micropore volumes (characterized by extensive BET surface areas), as noted in previous research^44^.

## Conclusion

This study successfully modeled carbon dioxide adsorption on carbon-based adsorbents using multilayer perceptron (MLP) and radial basis function (RBF) neural networks. Input variables such as BET surface, mesopore volume, micropore volume, temperature, and pressure were used in the models. After evaluating various training algorithms and activation functions, the Levenberg–Marquardt backpropagation algorithm with 'tansig' activation in hidden layers and linear output was identified as the optimal configuration for MLP models. The best MLP and RBF models achieved mean square error (MSE) values of 2.6293E−5 and 9.8401E−5, respectively. The MLP deep neural network with LM and BR training algorithms outperformed the RBF network, achieving a remarkable correlation coefficient of 0.9951 across a dataset of over 200 adsorbers. This study also revealed the significant influence of micropore volume at lower pressures and mesopore volume at higher pressures on CO_2_ uptake. The study has significantly contributed to the development of a comprehensive and efficient model for predicting carbon dioxide adsorption, leveraging prior research to establish a robust connection between the textural properties of adsorbents and operational conditions. This advancement enhances the ability to predict porous carbon CO_2_ uptake effectively.

### Supplementary Information

Supplementary Information.

## Data Availability

The datasets used and analysed during the current study available from the corresponding author on reasonable request.

## List of symbols

$M_{ads}^{{CO_{2} }}$: The amount of CO_2_ adsorbed
$$V_{micro}$$: Micropore volume
$$V_{meso}$$: Mesopore volume
$w^{i}$: Set of variables that need to be updated
$\nabla f(w^{i} )$: Gradient of the loss function f
$y^{0}$: Primary training direction vector
$$c^{i}$$: Conjugate parameter
$H$: Hessian matrix estimation
$$J$$: Jacobian matrix
$e$: Vector of network errors
$$g$$: Gradient
$$F$$: Objective function
$E_{D}$: Mean squared error function
$E_{W}$: Weight attenuation function

## List of Greek symbols

$$\eta^{i}$$: Learning rate
$$\mu$$: Scalar
$\alpha$: Hyper-parameters utilized to control the distribution of other parameters
$\beta$: Hyper-parameters utilized to control the distribution of other parameters
$\alpha_{\exp }$: Real experimental data points
$\alpha_{cal}$: Calculated data points

## List of acronym

DL: Deep learning
ANN: Artificial neural network
BP: Backpropagation
GD: Gradient descent
GDM: Gradient descent with momentum backpropagation
GDA: Gradient descent with adaptive learning rate backpropagation
GDX: Gradient descent with momentum and adaptive learning rate backpropagation
RP: Resilient backpropagation
CGF: Fletcher–Powell conjugate gradient
CGP: Polak–Ribiere conjugate gradient
CGB: Conjugate gradient with Powell–Beal restarts
SCG: Scaled conjugate gradient
LM: Levenberg–Marquardt
BFGS: Broyden–Fletcher–Goldfarb–Shanno algorithm
OSS: One step secant
BR: Bayesian regularization
MLP: Multi layer percepteron
RBF: Radial basis function
MSE: Mean square error
AARD%: Average absolute relative deviation percentage

## Terminology

Activation function: The activation function serves as a mathematical function situated between the input being received by the present neuron and the output transmitted to the subsequent layer.
Bias: Bias is a constant that aids the model in optimizing its fit to the provided data.
Neurons: Neurons serve as fundamental units within a complex neural network.
Epoch: Training involves inputs and outputs being fed into iterative steps, compared to target values for error calculation. Weights and biases are computed and adjusted at each epoch.
Weight: Describes the significance and strengths of the input to the Neurons
